# TIDEST: post-imputation differential expression testing for spatial transcriptomics data

**DOI:** 10.64898/2026.06.19.733432

**Published:** 2026-06-24

**Authors:** Lorenzo Testa, Jing Lei, Kathryn Roeder

**Affiliations:** 1 Department of Statistics & Data Science, Carnegie Mellon University, Pittsburgh PA, US; 2 L’EMbeDS, Sant’Anna School of Advanced Studies, Pisa, Italy; 3 Department of Computational Biology, Carnegie Mellon University, Pittsburgh PA, US

## Abstract

Spatial transcriptomics enables the study of tissue organization *in situ*, but many high-resolution platforms measure only a limited gene panel, leaving much of the transcriptome unobserved. Although deep learning methods can reconstruct missing genes from matched single-cell references, downstream differential expression (DE) analysis remains unreliable because prediction uncertainty and spatially structured sources of variation are typically ignored. These factors can bias effect estimates and inflate false discoveries. We present TIDEST, a framework for DE testing after spatial transcriptomic imputation. TIDEST uses information from measured genes to correct systematic errors in reconstructed expression and adjusts for latent spatial variation, such as tissue architecture or cell-type composition, that can create spurious differences between biological groups. Across extensive simulations, TIDEST maintains substantially better error control than existing approaches while preserving power. Applications to mouse brain, human glioblastoma, and human breast cancer data recover biologically meaningful DE signals that are missed or distorted by conventional analyses. TIDEST provides a principled framework for DE analysis on reconstructed spatial transcriptomes.

## Introduction

Spatial transcriptomics (ST) enables researchers to study tissue architecture and cellular interactions *in situ* [1], capturing information lost in traditional single-cell (scRNA-seq) or bulk RNA sequencing [2]. However, a fundamental trade-off persists between spatial resolution and genomic coverage. Sequencing-based platforms (10x Visium [3], Slide-seq [4]) provide transcriptome-wide measurements at lower spatial resolution, whereas imaging-based methods (10x Xenium [3], MERFISH [5], seqFISH [6–8]) achieve single-cell precision but capture only tens to hundreds of genes. Even sequencing-based platforms suffer from severe gene dropout due to limited sequencing depth, leaving many genes effectively unobserved. As a result, differential expression (DE) analysis across tissue regions, disease states and cellular niches – one of the primary goals of ST studies – is often performed on incomplete or sparsely observed transcriptomes.

To overcome these limitations, deep learning (DL) frameworks (such as Tangram [9] and gimVI[10]) reconstruct missing or dropout-affected gene expression using matched scRNA-seq references [9–22], aligning scRNA-seq cells to spatial locations based on shared expression patterns to “fill in” or denoise transcript abundances with high spatial accuracy. While these models excel at prediction, a critical hurdle remains for downstream analysis: current practice either ignores DL-imputed genes entirely or treats imputed values *as if* observed, ignoring the prediction uncertainty inherent in the process [23]. DE testing on reconstructed values is therefore often unreliable for two reasons. First, predictive algorithms introduce their own systematic biases that can be mistaken for biological signal. Second, the spatial proximity of similar cells violates the independence assumptions of standard tests, inflating significance and false-positive rates. Without accounting for both prediction error and spatial dependence, “discoveries” from imputed spatial data risk being statistical artifacts rather than biological findings.

Here we introduce *Testing for Imputed Differential Expression in Spatial Transcriptomics* (TIDEST), a framework for DE testing on reconstructed spatial transcriptomes that accounts for both prediction uncertainty and unobserved spatial confounding, such as tissue architecture, cell-type composition, or microenvironmental gradients. TIDEST first uses information from observed genes to correct systematic errors in imputed gene expression, reducing the impact of prediction uncertainty on downstream analyses. It then estimates and adjusts for latent spatial variation that may confound DE comparisons across biological conditions. In simulations mimicking the spatial architecture of real tissues, available methods such as SpaGCN [24], DESpace [25] and SpatialGEE [26], which ignore these issues, fail to control false positives under spatial autocorrelation and prediction bias, whereas TIDEST maintains stable error control while preserving high power. Applied to mouse brain, human glioblastoma, and human breast cancer ST data, TIDEST identifies DE genes that are obscured or misrepresented in conventional analyses, providing a rigorous framework for inference on reconstructed spatial transcriptomes.

## Results

### Overview of TIDEST

**Figure 1:**
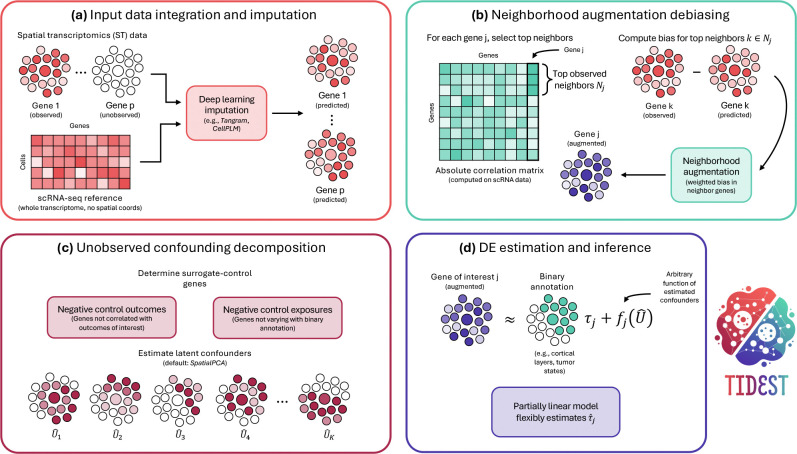
Overview of TIDEST. **(a)** TIDEST takes as input a spatial transcriptomics (ST) dataset, a matched scRNA-seq reference and the reconstructed transcriptome generated by an imputation model. The scRNA-seq reference provides transcriptome-wide information, whereas the ST data provide spatial localization. **(b)** TIDEST refines the reconstructed expression profiles using information from measured genes. For each target gene, the method identifies highly correlated observed genes and uses their prediction errors to correct systematic biases in the reconstructed expression. This produces an augmented expression profile that is less sensitive to errors introduced during imputation. **(c)** TIDEST identifies large-scale spatial patterns that may influence gene expression independently of the biological comparison of interest. Examples include anatomical structure, cell-type composition, microenvironmental gradients, and technical effects. These latent patterns are estimated from a set of control genes and summarized as a low-dimensional representation of spatial variation. **(d)** TIDEST performs differential expression testing between predefined biological groups while accounting for the estimated spatial variation. The output consists of effect estimates, confidence intervals, and significance measures for both observed and reconstructed genes, enabling transcriptome-wide DE analysis from spatially reconstructed transcriptomes.

TIDEST is a framework for transcriptome-wide differential expression testing in spatial transcriptomics datasets where some genes are reconstructed from a scRNA-seq reference. The workflow takes as input a ST dataset, a matched scRNA-seq reference, and the predicted spatial expression generated by an imputation model (Fig. 1a). TIDEST then refines the reconstructed expression profiles using information from measured genes (Fig. 1b), identifies latent spatial variation that may confound biological comparisons (Fig. 1c), and performs DE testing between predefined biological conditions (Fig. 1d). The output is a set of effect estimates, confidence intervals, and significance measures for both observed and reconstructed genes, enabling transcriptome-wide DE analysis while accounting for uncertainty introduced during spatial reconstruction.

The first stage addresses prediction uncertainty via a *debiasing* procedure (Fig. 1b) [27, 28]. Common practice treats imputed values ${\tilde{Y}}_{ij}$ for spatial coordinate i and gene j as true observations, ignoring the error introduced during reconstruction. TIDEST instead uses the observed residuals of highly correlated measured genes to construct an *augmented* outcome ${\overset{ˆ}{Y}}_{ij}$ for each target gene (see Methods), reducing systematic prediction errors and improving robustness to biases introduced by the underlying imputation model.

The second stage addresses unobserved confounding by estimating latent sources of variation from surrogate-control outcomes (Fig. 1c). In spatial transcriptomics, factors other than the biological comparison of interest can create apparent expression differences between groups. For example, anatomical structure, cell-type composition, microenvironmental gradients or technical effects may vary across a tissue and influence gene expression independently of the condition being studied [29]. TIDEST estimates these latent patterns (by default using SpatialPCA [30]) and incorporates them into downstream analyses, reducing the risk that such factors are mistaken for true differential expression signals.

The final stage performs DE testing while accounting for the estimated latent variation (Fig. 1d). TIDEST fits a partially linear model [31] relating the augmented outcomes to the biological condition of interest while flexibly adjusting for the estimated spatial confounders. Because inference is performed on the augmented outcomes rather than the raw predictions, TIDEST simultaneously addresses prediction uncertainty and unobserved spatial variation, enabling more reliable identification of differentially expressed genes across biological conditions.

### Simulation study provides strong support for TIDEST

**Figure 2:**
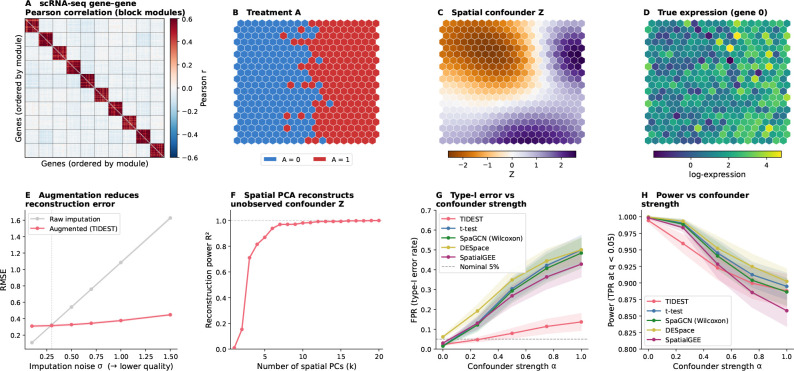
Simulation study validating TIDEST. **(a)** Block-diagonal Pearson correlation matrix of the synthetic scRNA-seq reference, computed from 2000 cells across 10 co-expression modules (200 genes). The block structure is the informative gene-gene correlation exploited by TIDEST augmentation strategy. **(b)** Binary treatment assignment A (two-region DGP, n=400 spots on a regular hexagonal grid). **(c)** Unobserved spatial confounder Z, drawn as a Gaussian random field with length-scale ℓ=0.3. In this simulation setting, Z is spatially smooth and independent of A, but its interaction with gene loadings $\beta_{j}$ induces systematic differences between treatment regions that naive comparisons absorb into the estimated treatment effect. **(d)** True log-expression of a representative DE gene, combining the treatment effect $\tau_{j}A_{i}$ and the confounder contribution $\alpha \beta_{j}Z_{i}$. **(e)** Reconstruction quality of the augmented pseudo-outcome. RMSE of the raw imputed prediction ($\tilde{Y}$, gray) and the augmented outcome ($\overset{ˆ}{Y}$, red) against the true latent expression Y, as a function of imputation noise $\sigma_{\text{imp}}$ (lower $\sigma_{\text{imp}}$ means better imputer). The dashed vertical line marks the crossover ($\sigma_{\text{imp}}\approx 0.3$) below which raw imputation is sufficient; above this threshold the augmented RMSE is bounded between 0.31 and 0.45 regardless of imputer quality. **(f)** Confounder reconstruction power. Coefficient of determination $R^{2}$ of Z as a function of the number of spatial PCA eigenvectors k retained in the confounder matrix U, computed on the illustration replicate. Three eigenvectors capture 71% of the confounder variance; 10 eigenvectors reach 98%. **(g)** Type-I error rate (FPR at BH q<0.05) vs α (50 replicates per α level). The dashed line marks the nominal 5% level. All competing methods inflate FPR steeply with confounding; TIDEST maintains substantially lower FPR across all settings. Shaded bands are 95% confidence intervals across replicates. **(h)** Power (TPR at BH q<0.05) vs confounder strength α (50 replicates per α level).

We validated TIDEST on fully synthetic data in which ground-truth treatment effects, spatial confounders, and imputation errors are known by design (see Methods). An example scRNA-seq reference, spatial treatment A, unobserved spatial confounder Z, and observed gene expression Y are shown in Fig. 2a-d. We compared TIDEST against a two-sample t-test and three literature-identified competitors [23]: SpaGCN[24] (Wilcoxon rank-sum test, also used in Seurat [32]), DESpace [25] (negative binomial generalized linear model), and SpatialGEE [26] (generalized estimating equations).

Reconstruction RMSE of the raw imputed predictions $\tilde{Y}$ and the augmented outcome $\overset{ˆ}{Y}$ against true latent expression as a function of imputation noise $\sigma_{\text{imp}}$ reveals a crossover at $\sigma_{\text{imp}}\approx 0.3$ (Fig. 2e). Raw imputation is sufficient below this point; above it – the typical operating regime for prediction methods – augmentation reduces RMSE by 40% at $\sigma_{\text{imp}}\approx 0.5$ and 72% at $\sigma_{\text{imp}}\approx 1.5$. Augmentation RMSE remains bounded between 0.31 and 0.45 regardless of imputer quality and decoupling testing fidelity from the imputation model. The learned confounder representation U approximates the unobserved field Z well (Fig. 2f): three eigenvectors explain $R^{2}=0.71$ of confounder variance, and as k increases $R^{2}=0.98$, yielding the near-complete deconfounding seen in Fig. 2g.

We examine the false positive rate (FPR) among null genes at BH-adjusted q<0.05 versus confounder strength α (50 replicates per setting) (Fig. 2g). At α=0, all methods are near the nominal 5% level except DESpace (FPR = 6.1%), slightly liberal due to anti-conservatism of the negative-binomial model on sparse counts. As α increases, all competitors inflate sharply: at α=1.0, the t-test and DESpace reach FPR = 50.0%, SpaGCN 48.4%, and SpatialGEE 42.8% – about ten times the nominal rate – while TIDEST attains FPR = 13.7%, a 3.6× improvement over the best competitor (SpatialGEE).

We next examine nominal power (TPR at BH q<0.05): at α=0 all methods achieve near-perfect power (≥ 99%), and at α=1.0 power converges toward 85–90% across methods (Fig. 2h). The apparent advantage of the t-test and DESpace[25] over TIDEST at high α is an artefact of inflated FPR – the BH threshold admits more false positives while true positives barely increase. This is evident from the area under the ROC curve (AUC; Supplementary Fig. B.1), which evaluates how well each method separates truly DE genes from null genes across all possible significance thresholds. At α=0 all methods achieve AUC ≈ 1, while at α=1.0 TIDEST achieves AUC = 0.932 versus 0.832 for DESpace (best competitor) and 0.811–0.821 for the rest – a 10 percentage-point advantage.

### TIDEST enables stable laminar profiling of the mouse neocortex

**Figure 3:**
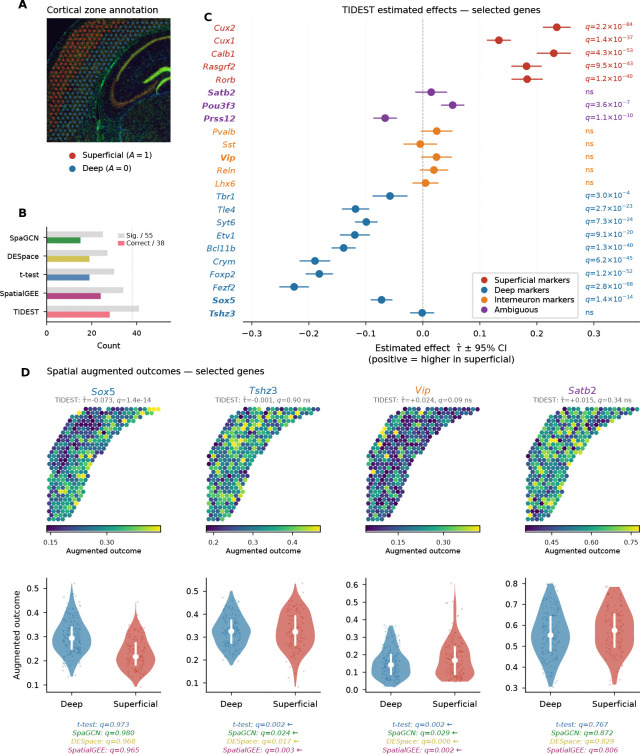
Mouse brain application. **(a)** Visium section of the adult mouse neocortex with cortical zone annotation overlaid as colored hexagonal spots (red: superficial layers L2/3/4, A=1; blue: deep layers L5/6, A=0; 297 spots total). **(b)** Method comparison summary. Grouped bars show, for each method, the number of genes called significant out of all 55 (gray) and the number that are both significant and correctly directioned out of the 38 genes with an established expected direction (colored). TIDEST achieves the highest correct count (28/38). **(c)** TIDEST estimated effects ($\overset{ˆ}{\tau }\pm 95\%\;CI$) for 23 selected genes. Color indicates gene category: red = superficial markers, blue = deep markers, orange = interneuron markers (negative controls), purple = ambiguous annotation. BH-adjusted q-values are shown to the right of each estimate. **(d, top row)** Spatial augmented outcome maps for four key genes (*Sox5*, *Tshz3*, *Vip*, *Satb2*). The subtitle reports the TIDEST estimate and BH-adjusted q-value. **(d, bottom row)** Deep vs. superficial augmented outcome distributions for the same four genes. Violins show the full distribution; the white bar marks the interquartile range; the white dot marks the median. BH-adjusted q-values for all four competing methods (SpaGCN, DESpace, t-test, SpatialGEE) are annotated below each violin. *Sox5*: TIDEST detects a deep signal ($q=1.4\times 10^{-14}$) that all competitors miss (q≥0.97). *Tshz3*: TIDEST returns a null result (q=0.90) while all competitors produce significant estimates in the wrong biological direction q≤0.024. *Vip*: an interneuron marker that may not differ by layer; TIDEST is non-significant (q=0.09) while all competitors produce significant results (q≤0.029). *Satb2*: expressed across both layers; all methods agree on non-significance.

We applied TIDEST to a Visium spatial transcriptomics section of the adult mouse neocortex [33], restricting to two annotated cortical zones: deep layers L5/6 (n=171 spots) and superficial layers L2/3/4 (n=126 spots), totaling 297 spots. The binary treatment is $A_{i}=1$ for superficial spots and $A_{i}=0$ for deep spots, so a positive τ indicates higher superficial expression (Fig. 3a). Although Visium nominally profiles the whole transcriptome, low per-spot sequencing depth produces extensive dropout, so we complemented the Visium data with a paired scRNA-seq reference (21697 cells) [34], projected onto the spatial domain via Tangram[9] to obtain denoised expression estimates for all genes. Because gene expression varies continuously across cortical depth [35, 36], ignoring this spatial structure risks confounding location effects with the layer contrast of interest (see Methods for pipeline details).

We assembled a panel of 55 curated cortical genes: 38 with an established expected direction (deep or superficial), 5 interneuron markers as negative controls (expected to show no layer bias), and 12 genes with unclear layer preference. TIDEST identified 41/55 (74.5%) as significantly differentially expressed (BH q<0.05); among the 38 directional genes, 28 were significant with the correct direction, 7 non-significant, and only 3 significant in the opposite direction (Fig. 3b). The strongest superficial signal was *Cux2* $\left(\overset{ˆ}{\tau }=+0.235,\;q=2.2\times 10^{-84}\right)$, a well-established upper-layer marker [37]; the strongest deep signal was *Fezf2* $\left(\overset{ˆ}{\tau }=-0.226,\;q=2.8\times 10^{-68}\right)$, required for corticospinal neuron specification [38, 39] (Fig. 3c; further examples in Supplementary Material).

We compared TIDEST against four competitors on the same gene panel (Fig. 3b; Supplementary Fig. C.3): TIDEST recovered the most correct-direction significant genes (28 of 38), versus SpatialGEE (24), t-test (19), DESpace (19), and SpaGCN (15). Two genes illustrate this advantage. *Sox5*, an established deep-layer marker [40, 41], is detected by TIDEST $\left(\overset{ˆ}{\tau }=-0.073,\;q=1.4\times 10^{-14}\right)$; Fig. 3d) but missed by all four competitors (q≥0.965). Conversely, *Tshz3*, a deep-layer gene [42], is correctly returned as non-significant by TIDEST (q=0.903), while all four competitors produce significant false positives in the *wrong* direction: t-test calls it superficial (q=0.002), as do SpatialGEE (q=0.003), DESpace (q=0.017), and SpaGCN (q=0.024) (Fig. 3d).

TIDEST also recovers signals aligning with recent transcriptomic evidence over older functional annotations (Fig. 3c); two further examples (*Pou3f3*, *Prss12*) are detailed in the Supplementary Material. *Satb2*, a nuclear matrix protein and transcription factor, was annotated as a superficial marker required for callosal projection neuron identity in L2/3 [43, 44], though its immunoreactivity has also been observed in L6, challenging this purely superficial interpretation [45, 46]. TIDEST returns a non-significant estimate (q=0.339; Fig. 3d), consistent with a gene that does not preferentially localize to either zone – a null result shared by all competing methods.

The clearest specificity advantage concerns interneuron markers, which are distributed across cortical layers and may not systematically differ between zones. *Vip*, a canonical interneuron marker [47], is called significant by every competitor (t-test q=0.002, SpatialGEE q=0.002, DESpace q=0.006, SpaGCN q=0.029; Fig. 3d), but TIDEST returns q=0.090 (non-significant). The remaining four interneuron markers were likewise non-significant in TIDEST (Supplementary Table C.1). These results may arise from unobserved confounding: apparent layer association detected by competing methods may reflect local interneuron clustering rather than robust laminar enrichment.

### TIDEST disentangles tumor-core and leading-edge expression programs in human glioblastoma

**Figure 4:**
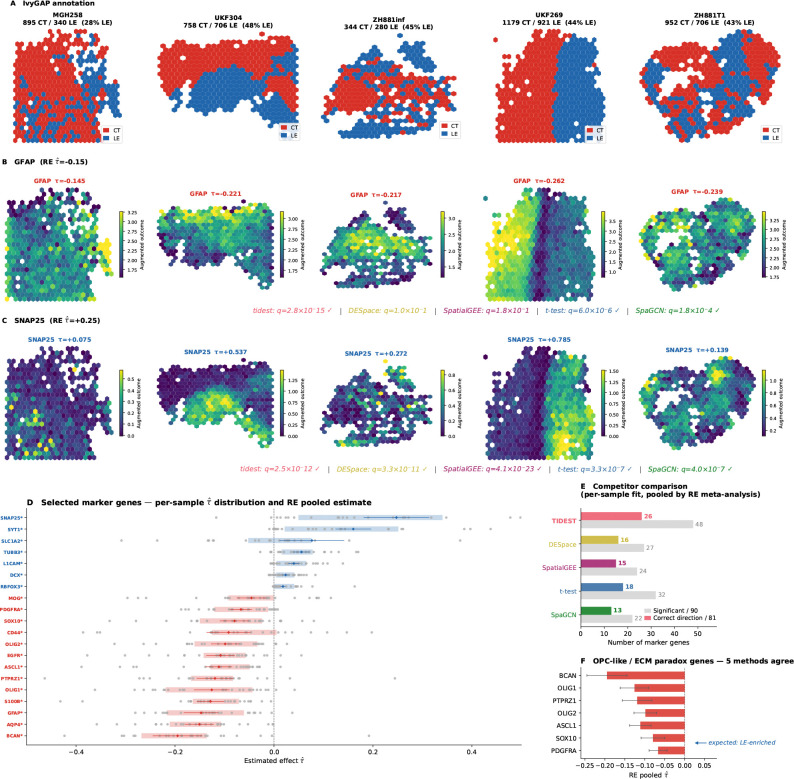
Human glioblastoma application. **(a–c)** IvyGAP histological annotation (**a**; CT in red, LE in blue) and augmented spatial expression maps of *GFAP* (**b**) and *SNAP25* (**c**) across five representative GBM sections (MGH258, UKF304, ZH881inf, UKF269, ZH881T1), illustrating the spatial cell-composition gradient that TIDEST disentangles from genuine tumor biology. Per-sample $\overset{ˆ}{\tau }$ values are shown above each map; pooled random-effects estimates are given in the row titles. Colored annotations below the maps report each method’s q-value for that gene, illustrating TIDEST’s power advantage on representative markers. **(d)** Per-sample $\overset{ˆ}{\tau }$ estimates (dots) and random-effects pooled estimates with 95% confidence intervals (diamonds and shaded bands) for a curated panel of marker genes, ordered by pooled $\overset{ˆ}{\tau }$ from strongly cellular-tumor (CT)-enriched (left, red) to strongly leading-edge (LE)-enriched (right, blue). **(e)** Number of the 90 panel genes called significant (BH q<0.05; gray bars) and, of the 81 genes with an *a priori* expected CT/LE direction, the number called significant with the correct sign (colored bars), for TIDEST and four competing methods (t-test, SpaGCN, DESpace, SpatialGEE), each fitted independently per sample across all 26 sections and pooled with the same DerSimonian-Laird random-effects meta-analysis. **(f)** Random-effects pooled $\overset{ˆ}{\tau }$ (with 95% confidence intervals) for the seven OPC-like and ECM “paradox” genes (*BCAN*, *OLIG1*, *PTPRZ1*, *OLIG2*, *ASCL1*, *SOX10*, *PDGFRA*). All seven are robustly CT-enriched ($\overset{ˆ}{\tau }<0$, BH $q<10^{-6}$ for every gene) and all five tested methods agree unanimously on the CT-enriched sign for every gene.

We applied TIDEST to a cohort of 26 IDH-wildtype GBM Visium sections spanning three independent patient cohorts (MGH, UKF, ZH) [48], fitting TIDEST independently to each section using the Ivy Glioblastoma Atlas Project (IvyGAP) annotation [49]. We focused on spots assigned to *cellular tumor* (CT; the proliferating tumor core) or *leading edge* (LE; the infiltrating margin into normal brain), with $A_{i}=1$ for LE and $A_{i}=0$ for CT, so a positive $\overset{ˆ}{\tau }$ indicates higher expression at the invasion front (Fig. 4a). Imputation used Tangram [9] with an external scRNA-seq reference from a disjoint cohort [50], used here to denoise dropout-affected counts. We assembled a curated panel of 90 genes (Supplementary Table D.2) covering GBM tumor cell states, neuronal markers, myelin proteins, invasion mediators, immune microenvironment, and metabolic markers. The CT-versus-LE contrast is a demanding test of spatial deconfounding: CT regions are densely packed with neoplastic cells, whereas LE regions mix infiltrating tumor cells with resident normal brain tissue, so any comparison ignoring this compositional gradient risks attributing cell-type density differences to tumor biology itself.

Of the 90 curated genes (all present in the Visium panel), TIDEST identified 48 as significantly differentially expressed in the random-effects meta-analysis (DerSimonian-Laird; BH q<0.05), with results consistent across the 3 cohorts. The strongest CT-enriched signals were the classical GBM astrocyte-state markers [51]: *AQP4* ($\overset{ˆ}{\tau }=-0.151,\;q=2.4\times 10^{-22}$, significant in 85% of samples), *S100B* ($\overset{ˆ}{\tau }=-0.128,\;q=4.0\times 10^{-18},\;92\%$), and *GFAP* ($\overset{ˆ}{\tau }=-0.147,\;q=2.8\times 10^{-15},\;73\%$), consistent with the high density of neoplastic astrocyte-like cells in the tumor core [52] (Fig. 4b). *EGFR* – amplified in ~40% of GBM – was robustly CT-enriched ($\overset{ˆ}{\tau }=-0.108,\;q=2.3\times 10^{-12},\;73\%$), as was *CD44* ($\overset{ˆ}{\tau }=-0.092,\;q=1.4\times 10^{-4},\;77\%$) [53].

The strongest positive-$\overset{ˆ}{\tau }$ signals were all normal-brain markers, led by the canonical synaptic vesicle proteins *SYT1* and *SNAP25* (*SYT1*: $\overset{ˆ}{\tau }=+0.160,\;q=5.7\times 10^{-17},\;73\%$; *SNAP25*: $\overset{ˆ}{\tau }=+0.247,\;q=2.5\times 10^{-12},\;77\%$; Fig. 4c), with several additional neuronal and astrocyte markers also LE-enriched (Supplementary Material). These findings confirm the IvyGAP annotation at the molecular level: GBM invasion proceeds by interdigitation of tumor cells with existing neurons and glia rather than wholesale displacement of normal tissue [50, 54]. The recovery of *L1CAM* – which mediates GBM cell migration along white matter tracts [54] – as a strong LE signal is notable, consistent with a role at the active invasion front rather than the tumor core.

We compared TIDEST against four published methods, each fitted independently on all 26 GBM sections and pooled with the same DerSimonian-Laird random-effects meta-analysis (Fig. 4e). For every method we report, on the same two denominators: how many of the 90 panel genes it calls significant (BH q<0.05), and how many of the 81 genes with an *a priori* expected CT/LE direction are both significant *and* correctly signed. TIDEST dominates on detection power: 48/90 and 26/81, versus 32/90 and 18/81 for the t-test, 27/90 and 16/81 for DESpace, 24/90 and 15/81 for SpatialGEE, and 22/90 and 13/81 for SpaGCN. TIDEST thus detects roughly 1.5–2× more significant genes than any competitor while maintaining comparable direction accuracy among significant calls, indicating that its power advantage reflects better-controlled unobserved confounding rather than a more permissive threshold. DESpace and SpatialGEE, which explicitly model spatial structure, modestly outperform the naive t-test and SpaGCN on direction accuracy (59% and 63% vs. 56% and 59%), underscoring that accounting for unobserved confounding drives the gains in this application.

Two gene classes exhibit a notable paradox: they were predicted from single-cell studies to be LE-enriched but are consistently identified as CT-enriched in the Visium data (Fig. 4f) [51]. The first comprises OPC- and NPC-like GBM state markers, including *ASCL1* ($\overset{ˆ}{\tau }=-0.111,\;q=8.9\times 10^{-15}$, significant in 88% of samples), *OLIG1* ($q=4.4\times 10^{-11},\;81\%$), *OLIG2* ($q=8.2\times 10^{-11},\;73\%$), *SOX10* ($q=5.0\times 10^{-7},\;73\%$), and *PDGFRA* ($q=2.6\times 10^{-8},\;58\%$). The second comprises invasion-associated ECM markers: *BCAN* (*Brevican*; $\overset{ˆ}{\tau }=-0.195,\;q=3.9\times 10^{-13},\;92\%$) and *PTPRZ1* ($\overset{ˆ}{\tau }=-0.119,\;q=1.6\times 10^{-9},\;88\%$). This paradox is likely explained by Visium’s 55, *μ*m spot resolution and the resulting mixing of tumor cells across spatial compartments (Supplementary Material). Consistent with this interpretation, all four methods recover the same CT-enriched sign for all seven genes (Supplementary Table D.3), suggesting that the discrepancy reflects a limitation of the underlying measurement technology rather than a method-specific effect.

### TIDEST identifies the marker programs of human breast cancer progression

**Figure 5:**
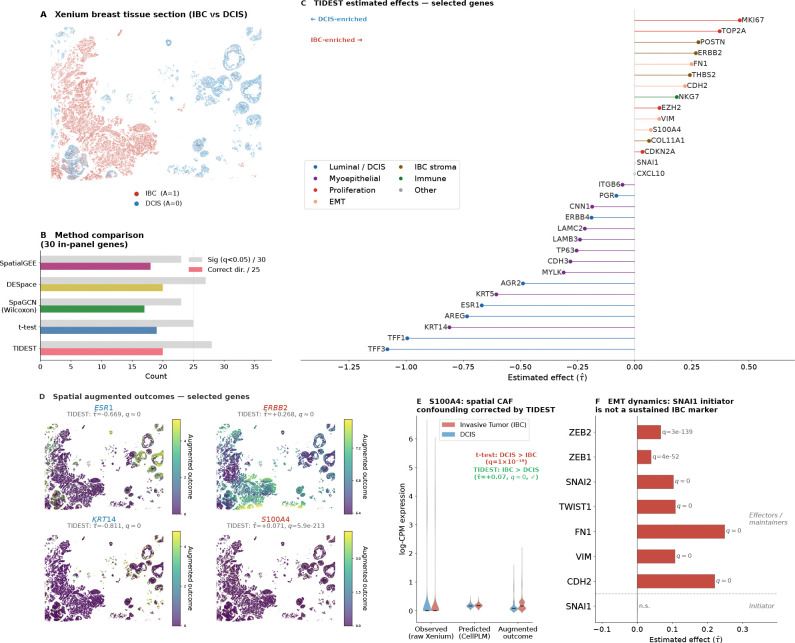
Human breast cancer application. **(a)** Xenium spatial transcriptomics section of human breast tissue (62755 cells). Each point is a single cell; red = IBC (A=1,n=28961); blue = DCIS (A=0,n=33794). **(b)** Method comparison on the 30 genes present in the observed Xenium panel. Grouped horizontal bars show, for each method, the number of genes called significant out of 30 (gray) and the number that are both significant and correctly directioned out of the 25 directional in-panel genes (red, TIDEST color). Dashed vertical line marks 25 (total directional genes). TIDEST and DESpace tie at 20/25 correct directions. **(c)** TIDEST estimated effects $\overset{ˆ}{\tau }$ for selected labeled marker genes, sorted by effect size (DCIS-enriched at left, IBC-enriched at right). Color encodes biological group (see legend). Dashed vertical line at $\overset{ˆ}{\tau }=0$. **(d)** Spatial augmented outcome maps for four key genes (*ESR1*, *ERBB2*, *KRT14*, *S100A4*). Gene name color encodes direction (red = IBC-enriched; blue = DCIS-enriched). Subtitle reports the TIDEST $\overset{ˆ}{\tau }$ and q-value. **(e)** *S100A4*: distributions of observed Xenium expression (left), CellPLM predicted expression (center), and augmented outcome (right), split by IBC (red) and DCIS (blue). Naive comparison using t-test on observed expression returns DCIS > IBC ($q=1\times 10^{-19}$ incorrect direction). TIDEST on the augmented outcome recovers the biologically expected IBC enrichment ($\overset{ˆ}{\tau }=+0.07,\;q\approx 0$). **(f)** EMT dynamics. *SNAI1* (initiator) is non-significant ($\overset{ˆ}{\tau }\approx 0,\;q=0.15$); all seven downstream EMT effectors and structural remodeling markers are significantly IBC-enriched, consistent with a tumor that has completed rather than is currently undergoing mesenchymal transition.

We applied TIDEST to a Xenium single-cell spatial transcriptomics dataset of human breast tissue containing both invasive breast cancer (IBC) and ductal carcinoma *in situ* (DCIS) regions in the same section [3], analyzing 62755 annotated locations (Fig. 5a), with $A_{i}=1$ for IBC and $A_{i}=0$ for DCIS, so a positive $\overset{ˆ}{\tau }$ indicates higher expression in invasive regions. Because the Xenium panel covers only 313 genes, we constructed the augmented outcome by imputing the full transcriptome via CellPLM [22], a pre-trained cell language model, using a matched scRNA-seq reference (27000 cells) [3]. IBC and DCIS cells arise in the same tissue but differ systematically in local microenvironment – IBC regions are surrounded by activated stroma with a disrupted basement membrane, while DCIS regions retain an intact myoepithelial layer – making unobserved confounding a primary concern for any naive group comparison.

We assembled a panel of 68 curated genes spanning IBC proliferation and invasion markers, DCIS luminal markers, myoepithelial keratins, EMT effectors, immune infiltrate markers, and contested genes; 25 have a well-established expected direction in the IBC-versus-DCIS contrast. TIDEST identified 65/68 (95.6%) as significantly differentially expressed (BH q<0.05) and recovered the correct direction for 20/25 known directional genes (Fig. 5b). The strongest DCIS signals were canonical luminal markers: *TFF3* ($\overset{ˆ}{\tau }=-1.08,\;q\approx 0$), *TFF1* ($\overset{ˆ}{\tau }=-1.00,\;q\approx 0$), *AREG* $\left(\overset{ˆ}{\tau }=-0.73,\;q\approx 0\right)$, *AGR2* ($\overset{ˆ}{\tau }=-0.49,\;q\approx 0$), and *ESR1* ($\overset{ˆ}{\tau }=-0.67,\;q\approx 0$). The strongest IBC signals were proliferation and invasion drivers: *MKI67* ($\overset{ˆ}{\tau }=+0.46,\;q\approx 0$), *TOP2A* ($\overset{ˆ}{\tau }=+0.37,\;q\approx 0$), *ERBB2* ($\overset{ˆ}{\tau }=+0.27,\;q\approx 0$), *POSTN* ($\overset{ˆ}{\tau }=+0.28,\;q\approx 0$), and the mesenchymal marker *FN1* ($\overset{ˆ}{\tau }=+0.25,\;q\approx 0$) [55] (Fig. 5c,d).

We compared TIDEST against competing methods on 30 genes present in the observed Xenium panel (Fig. 5b). Among the 25 directional in-panel genes, TIDEST recovered 20 with the correct direction – tied with DESpace (20) and ahead of the t-test (19), SpatialGEE (18), and SpaGCN (17) – while also detecting the most total significant genes (28/30). *S100A4*, a cancer-associated fibroblast (CAF) marker linked to tumor invasion [56] and expected IBC-enriched, is called significant by the t-test in the *wrong* direction ($q=1\times 10^{-19}$), because CAFs interdigitated with DCIS ducts are absorbed into the group mean under naive comparison; TIDEST corrects for this and recovers the expected IBC enrichment ($\overset{ˆ}{\tau }=+0.071,\;q=6\times 10^{-213}$; Fig. 5e). *NKG7* provides a further example of this gain (Supplementary Material).

*SNAI1*, the canonical initiator of epithelial-to-mesenchymal transition (EMT) in breast cancer [57], transcriptionally represses E-cadherin to trigger the EMT program. Yet TIDEST finds *SNAI1* non-significant ($\overset{ˆ}{\tau }=+0.001,\;q=0.15$; Fig. 5f), consistent with its expression being transient – upregulated to initiate invasion but not maintained in established IBC cells. By contrast, all seven EMT-associated transcription factors and mesenchymal/remodeling markers of *SNAI1* are significantly IBC-enriched: *CDH2* ($\overset{ˆ}{\tau }=+0.22,\;q\approx 0$), *VIM* ($\overset{ˆ}{\tau }=+0.11,\;q\approx 0$), *TWIST1* $\left(\overset{ˆ}{\tau }=+0.11,\;q\approx 0\right)$, *FN1* ($\overset{ˆ}{\tau }=+0.25,\;q\approx 0$), *ZEB1* ($\overset{ˆ}{\tau }=+0.040,\;q=4\times 10^{-52}$), and *ZEB2* ($\overset{ˆ}{\tau }=+0.067,\;q=3\times 10^{-139}$). This pattern – initiator null, effectors strongly positive – may characterize a tumor that has completed rather than is currently undergoing mesenchymal transition.

Some results warrant biological interpretation rather than simple validation. The keratins *KRT5* $\left(\overset{ˆ}{\tau }=-0.61\right)$ and *KRT14* ($\overset{ˆ}{\tau }=-0.81$) are strongly DCIS-enriched; although often listed as basal-type markers of aggressive breast cancer, in this spatial contrast they instead reflect the intact myoepithelial layer surrounding DCIS ducts, lost upon basement membrane breach in IBC [58, 59] – a result confirmed by all four competitors and consistent with additional myoepithelial markers and immune genes (see Supplementary Material).

## Discussion

Spatial transcriptomics has transformed our ability to study tissue organization by preserving the spatial context of molecular measurements. However, growing reliance on computational reconstruction has exposed a fundamental disconnect between prediction and inference. While modern deep learning models can accurately impute unmeasured genes using matched single-cell references, downstream analyses typically treat reconstructed expressions as if they were directly observed. This practice ignores both prediction uncertainty and the complex spatial structure of biological tissues, potentially leading to biased effect estimates and inflated false discovery rates. To address this challenge, we developed TIDEST, a framework for valid differential expression analysis following spatial transcriptomic imputation that can be applied to reconstructed data generated by arbitrary DL models.

Across extensive simulations and three real-data applications spanning mouse neocortex, glioblastoma, and breast cancer, TIDEST demonstrated that accounting jointly for prediction uncertainty and unobserved spatial confounding is critical for reliable post-imputation inference. Compared with existing approaches, TIDEST consistently achieved superior control of false positives while maintaining competitive statistical power. Importantly, it identified biologically coherent differential expression patterns that were missed, attenuated, or incorrectly attributed by methods that ignored one or both sources of bias. These results highlight that accurate reconstruction alone is insufficient for valid downstream discovery and that principled uncertainty quantification must accompany predictive modeling in spatial omics analyses.

Several limitations warrant consideration. First, TIDEST depends on the availability of an appropriate single-cell reference, and performance may decline when the reference fails to capture the full cellular diversity represented in the spatial dataset. Second, the augmentation procedure assumes that prediction errors can be partially inferred from highly correlated observed genes; this assumption may be less reliable in settings with weak gene–gene correlation structure. Third, the current framework focuses on binary differential expression testing. Extending the methodology to continuous exposures, multiple experimental conditions, temporal trajectories, and hierarchical study designs represents an important direction for future research. Finally, variation in cell-type composition is a major determinant of spatial expression heterogeneity and may confound differential expression analyses. In the current implementation, such effects are absorbed into the estimated latent factors, enabling adjustment for broad compositional gradients without explicitly modeling cell identity. Nevertheless, integrating cell-type-specific information directly into the inferential framework may further improve power and interpretability.

More broadly, the combination of prediction-powered augmentation, latent confounder estimation, and orthogonalized inference provides a general strategy for valid statistical analysis of reconstructed molecular measurements. Beyond differential expression testing, these principles may be extended to gene-set enrichment analysis, co-expression network inference, detection of spatially variable genes, and causal analyses in spatial omics. The framework may also prove valuable for emerging modalities, including spatial proteomics and multimodal imaging technologies, where computational reconstruction is increasingly used to enhance measurement resolution and coverage. As spatial profiling technologies continue to evolve, reconstruction models will play an increasingly central role in biological discovery. Yet predictive accuracy alone does not guarantee valid inference. TIDEST establishes a foundation for principled analysis of reconstructed spatial molecular landscapes, helping bridge the gap between prediction and discovery in spatial omics.

## Methods

### Details on TIDEST

*Testing for Imputed Differential Expression in Spatial Transcriptomics* (TIDEST) is a multi-stage framework designed to perform rigorous, spatially aware hypothesis testing on imputed spatial transcriptomics data. The pipeline addresses the dual challenges of prediction uncertainty – where downstream workflows naively treat noisy, deep learning-generated imputations as ground truth observations – and spatial autocorrelation, which violates standard sample independence assumptions and induces severe false-positive inflation in traditional statistical tests. To restore valid inference, TIDEST processes imputed expression matrices through three sequential modules: neighborhood augmentation debiasing, spatial gradient decomposition via surrogate-control outcomes, and orthogonalized DE effect estimation using partially linear models (PLMs).

To calibrate the systematic biases inherent in deep learning-based spatial reconstructions, TIDEST constructs a debiased augmented outcome ${\overset{ˆ}{Y}}_{ij}$ for each spatial coordinate i and target gene j. Rather than conducting downstream inference directly on the raw imputed values ${\tilde{Y}}_{ij}$, the framework leverages the empirical prediction residuals from a designated genomic neighborhood $N_{j}$ consisting of highly correlated, physically measured anchor genes. For a given target gene j, $N_{j}$ is populated by the most highly correlated, spatially observed genes (default: top 5 genes), where the feature-feature correlations ${\tilde{\sigma }}_{jk}$ are estimated from a scRNA-seq reference matching the biological system. Let $Y_{ik}$ denote the true observed expression of neighbor gene $k\in N_{j}$ at location i, and let ${\tilde{Y}}_{ik}$ be its corresponding model imputation. The debiased augmented outcome for the target gene is defined as:

(1) $${\overset{ˆ}{Y}}_{ij}={\tilde{Y}}_{ij}+\frac{1}{\left|N_{j}\right|}\sum_{k\in N_{j}}{\tilde{\sigma }}_{jk}\left(Y_{ik}-{\tilde{Y}}_{ik}\right).$$

By averaging the measured errors of observed spatial anchors, this procedure dynamically reduces the bias of unmeasured or sparsely captured transcripts exploiting information from ground-truth signals.

Spatially resolved transcriptomics data exhibit pervasive spatial autocorrelation, where large-scale tissue architectures (e.g., anatomical layering or tumor-microenvironment gradients) create continuous spatial patterns. When these structural variations correlate with a binary condition or tissue boundary, they act as unobserved confounders U, inflating Type-I error rates by violating standard independence assumptions. TIDEST addresses this by implementing an empirical spatial whitening procedure anchored on surrogate-control outcomes [60]. This strategy is closely related to surrogate variable analysis, where latent factors estimated from control features are used to account for unobserved variation [29]. In the present setting, these latent factors may represent spatial structure, cellular composition, technical artifacts, or other unmeasured sources of heterogeneity. To safely isolate local biological variation from confounding tissue-level trends, TIDEST scans the transcriptomic panel to identify a set of surrogate-control genes that satisfy two precise operational constraints:

- **Negative control outcome.** The empirical Pearson correlation between the target genes of interest and the candidate surrogate controls computed on the scRNA-seq reference falls below a threshold $r_{NCO}$.
- **Negative control exposure.** The spatial variability of the candidate surrogate controls – quantified by their coefficient of variation, defined as 100*γ/μ, where γ is the standard deviation and μ is the mean of a given spatial gene expression – falls below a threshold $r_{\text{NCE}}$.

These joint conditions guarantee that the selected surrogate-control genes are empirically decoupled from both the target biological outcomes and the primary treatment exposure. Once this control set is established, its spatial expression profile is utilized to reconstruct a low-dimensional representation of the latent confounding field $\overset{ˆ}{U}$. By default, TIDEST utilizes SpatialPCA to extract these latent principal components via a kernelized eigendecomposition [30], though alternative spatial dimension reduction techniques can be seamlessly integrated into the pipeline (e.g., StaNMF[61]).

In the final stage, TIDEST decouples the true treatment effect from large-scale spatial dependencies by mapping the augmented outcomes into a Robinson partially linear model (PLM) [31]. The conditional expectation of the debiased outcome is modeled as:

(2) $$E\left[{\overset{ˆ}{Y}}_{ij}|A_{i},{\overset{ˆ}{U}}_{i},X_{i}\right]=A_{i}\tau +f\left({\overset{ˆ}{U}}_{i},X_{i}\right),$$

where τ represents the core estimand of interest (the treatment effect of the binary condition A), $\overset{ˆ}{U}$ is the matrix of estimated confounders, and X captures additional spot-level covariates of interest (e.g., log-transformed library size). f(⋅) represents a flexible and unknown function mapping the spatial and technical confounders to expression levels.

By default, TIDEST utilizes random forests within a cross-fitting framework to flexibly estimate the nuisance functions in Equation 2. Crucially, this formulation yields an orthogonalized score function, guaranteeing that $\overset{ˆ}{\tau }$ achieves asymptotic normality and valid confidence intervals even when the non-parametric nuisance estimators or latent spatial components are estimated at slower, non-parametric rates [60].

### Simulation study design

We generated synthetic datasets consisting of n=300 spots on a regular two-dimensional grid, p=200 genes partitioned into 10 non-overlapping co-expression modules (20 genes each). We generated the binary treatment $A_{i}$ using the “two-region” DGP: treatment is a clean rectangular split – left half of the grid is assigned with A=0, right half has A=1. This is the most adversarial setting for naive methods because the spatial confounder Z (a smooth Gaussian field) correlates strongly with the treatment boundary. In the Supplementary Material, we explore additional DGPs. The true latent log-expression for gene j at spot i follows

(3) $$Y_{ij}=1+A_{i}\tau_{j}+\alpha Z_{i}\beta_{j}+\varepsilon_{ij},$$

where $\tau_{j}∼N\left(\tau_{\text{level}},0.1\tau_{\text{level}}\right)$ for the $p_{\text{DE}}=50$ differentially expressed genes, with random sign, and $\tau_{j}=0$ for the $p-p_{\text{DE}}=150$ null genes; $\beta_{j}∼N(0,1)$ are gene-specific confounder loadings; $Z_{i}∼GP\left(0,K_{\ell }\right)$ is a spatially smooth Gaussian random field with length-scale ℓ=0.3 that is independent of A;α∈{0,0.25,0.50,0.75,1.0} scales the confounder contribution; and $\varepsilon_{ij}∼N(0,1)$. Observed counts are drawn as $C_{ij}∼Poisson\left(exp\left(Y_{ij}\right)\right)$.

The imputation model is parameterized as

(4) $${\tilde{Y}}_{ij}=Y_{ij}+\sigma_{imp}l_{j}^{⊤}\eta_{i}+\epsilon_{ij},$$

where $\eta_{i}∼N\left(0,I_{M}\right)$ introduces module-structured noise, $l_{j}$ is the module loading vector of gene j (one entry is drawn from Uniform(0.5, 1.5), measuring the strength of its module membership; all the other entries are zero), and $\sigma_{imp}\in \{0.1,0.5,1.0,1.5\}$ parameterizes imputation quality (smaller means better). We also generate a synthetic single-cell reference of 2000 cells sharing the same module structure, so the Pearson correction exploits a realistic gene-gene correlation matrix.

We compared TIDEST against four spatial DE methods, all receiving identical observed count matrices and spatial coordinates: t-test (Welch two-sample t-test on log1p-normalized counts); SpaGCN[24] (Wilcoxon rank-sum test between spatial domains); DESpace [25] (negative-binomial model with spatial domain as a factor); and SpatialGEE [26] (generalized estimating equation with Poisson family and k-means working correlation structure).

In TIDEST pipeline, unobserved confounders were estimated using SpatialPCA[30] by truncated eigendecomposition of the Gaussian spatial kernel matrix retaining the top 20 eigenvectors. Eigenvectors with absolute Pearson correlation |r|>0.5 with the treatment A were dropped from the confounder matrix U prior to fitting. The PLM was fitted with two cross-fitting folds and 100 regression trees per nuisance estimator.

The main simulation grid crossed five confounder levels α∈{0,0.25,0.50,0.75,1.0} with three DGP variants and fixed $\tau_{\text{level}}=1.0$, $\sigma_{\text{imp}}=0.5$, yielding 15 settings; each was replicated 50 times. The DGP illustration panels (**A**-**D**) used n=400 spots on a hexagonal grid for visual clarity. The augmentation sweep (panel **E**) varied $\sigma_{\text{imp}}$ independently across six levels {0.1, 0.3, 0.5, 0.7, 1.0, 1.5} with 100 replicates per level, holding α=0 to isolate reconstruction quality from confounding. Extended results including all α levels and DGP-variant breakdowns are provided in the Supplementary Material.

### Real-data processing

#### Mouse brain data

We used a mouse neocortex Visium section dataset [33], restricting to spots annotated as “Cortex_1” (deep layers L5/6, n=171) or “Cortex_3” (superficial layers L2/3/4, n=126; 297 spots total). We assembled a panel of 55 curated layer-marker genes comprising 15 genes expected to be superficial-enriched, 23 deep-enriched, and 17 additional markers (including cell-type markers and negative controls); expected directions were assigned from published layer-specific expression atlases [35, 62]. The scRNA-seq reference was an adult mouse cortex dataset [34] (21697 cells), normalized to 10^4^ counts. Tangram[9] was fitted with RNA-count-based density prior; imputed expression was Pearson-augmented using the top-5 nearest-neighbors. SpatialPCA[30] was run retaining 10 principal components; one PC with absolute correlation |ρ|=0.779 with the treatment A was dropped by the correlation screen (threshold |ρ|>0.5), leaving nine SpatialPCA components as an estimate of unmeasured confounding. Log library size was appended as a covariate. The Robinson PLM was fitted with two cross-fitting folds and 200 regression trees per nuisance estimator (random-forest implementation). The four competing methods (t-test, SpaGCN Wilcoxon, DESpace, and SpatialGEE) were applied with identical settings to the simulation study, operating on the log1p-normalized observed Visium counts. All methods received the same 55 marker genes, and FDR control was applied independently within each method using the Benjamini-Hochberg procedure.

#### Human glioblastoma data

We analyzed 26 IDH-wildtype GBM Visium spatial transcriptomics sections from three patient cohorts [48]: the Massachusetts General Hospital (MGH) cohort (n=1), the University of Freiburg (UKF) cohort (n=13), and the University Hospital Zurich (ZH) cohort (n=12). Each section is annotated with IvyGAP histological regions [49]; we retained only spots annotated as cellular tumor (CT) or leading edge (LE), discarding spots with other IvyGAP annotations. We assembled a curated panel of 90 genes (predicted) representative of the major GBM molecular programs, normal brain cell types, immune microenvironment, and metabolic states. As TIDEST requires a single-cell reference for imputation, we used the human GBM scRNA-seq dataset [50] (3589 cells from a disjoint cohort of patients). The single-cell reference was normalized to 10^4^ counts. For Tangram[9] training we selected the top 100 marker genes per reference cell-type, using the reference cell-type labels as the grouping variable. Tangram was fitted in cells mode with an RNA-count-based density prior.

The augmented outcome for each spot i was constructed using the Pearson correlation correction with top-5 neighbors. The Pearson correlation matrix was computed once from the scRNA-seq reference and shared across all 26 samples. We applied per-spot total-count scaling to align the Tangram imputed expression to the observed Visium count scale before log-transformation. For each sample, we selected confounder genes for SpatialPCA as those with coefficient of variation $r_{NCE}<0.5$ in the augmented outcomes and Pearson correlation $\left|r_{NCO}\right|<0.3$ with any panel gene in the scRNA-seq reference. The top 2000 such genes ranked by spatial variance were used to fit SpatialPCA with 50 principal components. For each sample, SpatialPCA PCs with correlation with the treatment greater than 0.5 were excluded from the confounder matrix before fitting the PLM. Log-library size was appended as an additional covariate. We fitted the PLM independently for each sample using 5-fold cross-fitting and random forests with 200 trees. Per-sample *p*-values were adjusted by the Benjamini-Hochberg procedure within each sample (q<0.05).

Cross-sample meta-analysis used DerSimonian-Laird random-effects inverse-variance weighting to account for between-tumor heterogeneity, followed by a second round of BH correction over the 90 panel genes. The between-study variance ${\overset{ˆ}{\tau }}^{2}$ was estimated as ${\overset{ˆ}{\tau }}^{2}=max(0,(Q-(k-1))/c)$, where $Q=\sum_{i}w_{i}{\left({\overset{ˆ}{\tau }}_{i}-{\overset{ˆ}{\tau }}_{FE}\right)}^{2}$ is Cochran's heterogeneity statistic, k=26, and $c=\sum_{i}w_{i}-\sum_{i}w_{i}^{2}/\sum_{i}w_{i}$ with $w_{i}=1/{\overset{ˆ}{\sigma }}_{i}^{2}$.

#### Human breast cancer data

We used a Xenium FFPE spatial transcriptomics section of human breast tissue [3] containing both invasive breast cancer and ductal carcinoma *in situ* in the same specimen (62755 cells, 313-gene panel). Cluster annotations were taken from the companion single-cell reference [3]. The binary treatment was defined as $A_{i}=1$ for cells annotated as “Invasive_Tumor” or “Prolif_Invasive_Tumor” (IBC, n = 28961) and $A_{i}=0$ for cells annotated as “DCIS_1” or “DCIS_2” (DCIS, n=33794). We assembled a panel of 68 curated marker genes spanning IBC proliferation and invasion drivers, DCIS luminal markers, myoepithelial keratins, EMT effectors and maintainers, immune infiltrate markers, and contested genes; 25 genes have a well-established expected direction in the IBC-versus-DCIS contrast, assigned from published IBC, DCIS, and myoepithelial expression studies [58, 63, 64]. The expected direction for *KRT5* and *KRT14* was set to DCIS, as these myoepithelial markers reflect the intact basement membrane present in DCIS rather than the basal subtype designation used in gene-panel studies. Genes with unclear expected directions due to ER/HER2-status dependence (e.g., *EGFR*, *FOXA1*, *MLPH*, *GATA3*) were excluded from the directional evaluation.

Because the Xenium panel covers only 313 genes, we constructed the augmented outcome using CellPLM [22], a pre-trained Transformer-based cell language model. The matched single-cell reference (27000 cells, 18085 genes) was normalized to 10^4^ counts per cell and log1p-transformed before input to CellPLM; the same normalization was applied to the Xenium ST data. CellPLM outputs expression in log-CPM space, so the Pearson correction is applied directly as the difference between observed and predicted log-CPM, without additional scaling. The top-5 nearest-neighbor Pearson correction was applied to construct the augmented outcome. Confounder genes were selected using coefficient of variation threshold $r_{NCE}<1.5$ and marker correlation threshold $\left|r_{NCO}\right|\leq 0.3$, retaining 2000 genes; the higher $r_{NCE}$ threshold (compared to the Visium applications) reflects the greater single-cell-level expression variability in Xenium data.

Due to the large cell count, fitting SpatialPCA directly would require constructing a 62755 × 62755 kernel matrix, which exceeds available memory. We instead fitted SpatialPCA on a stratified subsample of 10000 cells (5000 per group), retaining 50 principal components, and then projected all 62755 cells into the fitted SpatialPCA space via Nyström kernel extension. No SpatialPCA component with absolute Pearson correlation |ρ|>0.5 with the treatment A was found, so none was excluded from the confounder matrix; log library size (log-CPM total per cell) was appended as a covariate. The PLM was fitted with 2 cross-fitting folds and 200 regression trees per nuisance estimator, retaining 50 SpatialPCA components (after the correlation screen) as confounders. Sandwich standard errors were computed from the cross-fitted residuals.

The four competing methods were applied to the 30 genes present in the observed Xenium panel, operating on log-CPM-normalized Xenium counts. For DESpace, 13 cells with zero total counts across the marker panel were filtered before constructing the SpatialExperiment object, as the calcNormFactors step in edgeR produces undefined normalization factors for zero-library cells. For SpatialGEE, a stratified subsample of 20000 cells (10000 per group) was used, as GEE estimation scales super-linearly with the number of observations. FDR control was applied within each method using the Benjamini-Hochberg procedure. TIDEST additionally tested the 38 genes accessible only through CellPLM augmentation; these were not evaluated for competitors.

## Supplementary Material

Supplement 1

## Data Availability

Mouse brain application data are available directly from the squidpy package [65] through the built-in functions datasets.visium_fluo_adata_crop() (10x Visium) and datasets.sc_mouse_cortex() (scRNA-seq). Human glioblastoma application spatial data (10x Visium) are available under the accession number GSE237183 (GEO) and also stored on Zenodo at https://doi.org/10.5281/zenodo.8105466. Single-cell reference data for the human glioblastoma application are available under the accession number GSE84465 (GEO). Human breast cancer application data are available under the accession code GSE243280 (GEO) and also stored on 10x Genomics website at https://www.10xgenomics.com/products/xenium-in-situ/preview-dataset-human-breast. All code to reproduce the entire analysis pipeline, as well as code containing TIDEST package, is available at https://github.com/testalorenzo/tidest.
