# Accurate prediction in reconstructed spatial transcriptomes does not ensure valid biological discovery

**DOI:** 10.64898/2026.06.19.733432

**Published:** 2026-08-10

**Authors:** Lorenzo Testa, Jing Lei, Kathryn Roeder

**Affiliations:** 1 Department of Statistics & Data Science, Carnegie Mellon University, Pittsburgh PA, US; 2 L’EMbeDS, Sant’Anna School of Advanced Studies, Pisa, Italy; 3 Department of Computational Biology, Carnegie Mellon University, Pittsburgh PA, US

## Abstract

Spatial transcriptomics is increasingly extended by computational reconstruction of unmeasured genes from matched single-cell RNA-sequencing references, enabling transcriptome-wide analyses from targeted or sparse assays. Yet downstream analyses typically treat reconstructed expression *as* experimentally observed, overlooking prediction error and latent spatial variation that can misattribute tissue architecture to biological regulation. Here we introduce TIDEST, a framework for statistically valid inference on reconstructed spatial transcriptomes. TIDEST calibrates reconstructed expression using measured genes and adjusts for latent spatial variation before differential expression analysis. Across realistic spatial tissue simulations, TIDEST controls false discoveries where existing approaches fail, while preserving power. Across mouse and human brain, glioblastoma and breast cancer, TIDEST changes biological interpretation by correcting misleading differential-expression calls and revealing disease-associated transcriptional programs obscured by spatial confounding. Our results show that prediction accuracy alone is insufficient for reliable biological discovery and establish valid statistical inference as an essential component of reconstructed spatial transcriptomics.

## Introduction

Spatial transcriptomics has transformed the study of tissue biology by measuring gene expression directly within intact tissues, preserving the spatial organization of cells and their local microenvironments [1]. By linking transcriptional programs to tissue architecture, it has become a powerful tool for identifying cellular niches, dissecting disease-associated microenvironments and characterizing developmental processes that cannot be resolved by bulk or dissociated single-cell RNA sequencing [2]. Yet current platforms face an inherent trade-off between spatial resolution and transcriptome coverage. Sequencing-based methods such as 10x Visium [3] and Slide-seq [4] profile thousands of genes but at limited spatial resolution, whereas imaging-based technologies such as 10x Xenium [3], MERFISH [5] and seqFISH [6–8] achieve single-cell or subcellular resolution while measuring only a targeted subset of transcripts. Even transcriptome-wide platforms suffer substantial gene dropout at low sequencing depth, leaving many biologically relevant genes incompletely observed. These limitations complicate one of the central goals of spatial studies: identifying genes whose expression differs across tissue regions, cellular neighborhoods and disease states.

To overcome incomplete coverage, a growing class of deep learning methods reconstructs unmeasured or sparsely observed expression by integrating spatial data with matched single-cell RNA-sequencing references [9–22]. Approaches such as Tangram [10] and gimVI [22] exploit shared transcriptional structure to infer transcriptome-wide spatial expression, substantially expanding the scope of downstream analyses, and have become widely used both for interpreting targeted imaging assays and for recovering expression lost to technical dropout. Reconstructed values, however, are predictions rather than direct molecular measurements. Current differential expression analyses typically either discard reconstructed genes or analyze predicted expression as though it were observed, overlooking the uncertainty introduced during reconstruction [23]. Compounding this, neighboring locations often share transcriptional programs driven by tissue architecture, cell-type composition and microenvironmental gradients, so apparent differences between biological groups may reflect where cells sit rather than the condition of interest. Together, prediction uncertainty and this latent spatial structure can produce misleading expression differences and erode confidence in the resulting biology. As a result, no existing method supports reliable differential expression analysis on reconstructed spatial transcriptomes.

Here we introduce *Testing for Imputed Differential Expression in Spatial Transcriptomics* (TIDEST), a framework that enables reliable differential expression analysis on reconstructed spatial transcriptomes. Unlike existing methods, which either discard reconstructed genes or treat predicted expression as if it were directly observed, TIDEST first calibrates reconstructed expression using measured transcripts to reduce systematic prediction error. It then estimates and adjusts for latent spatial variation arising from tissue architecture and cellular organization before testing for differential expression, providing a statistically principled foundation for inference. In realistic simulations of spatial tissue architecture, widely used methods including SpaGCN [24], DESpace [25] and SpatialGEE [26] fail to maintain appropriate false-positive rates when reconstruction error or spatial structure is present, whereas TIDEST achieves reliable error control while preserving power. Applied to mouse and human brain, glioblastoma and breast cancer, TIDEST corrects misleading differential-expression calls and reveals spatial genetic and disease-associated transcriptional programs across diverse biological settings – including cortical lamination, genetic risk for neuropsychiatric traits, glioblastoma cell states, and breast cancer invasion – that are missed or mischaracterized by conventional analyses.

## Results

### Overview of TIDEST

**Figure 1:**
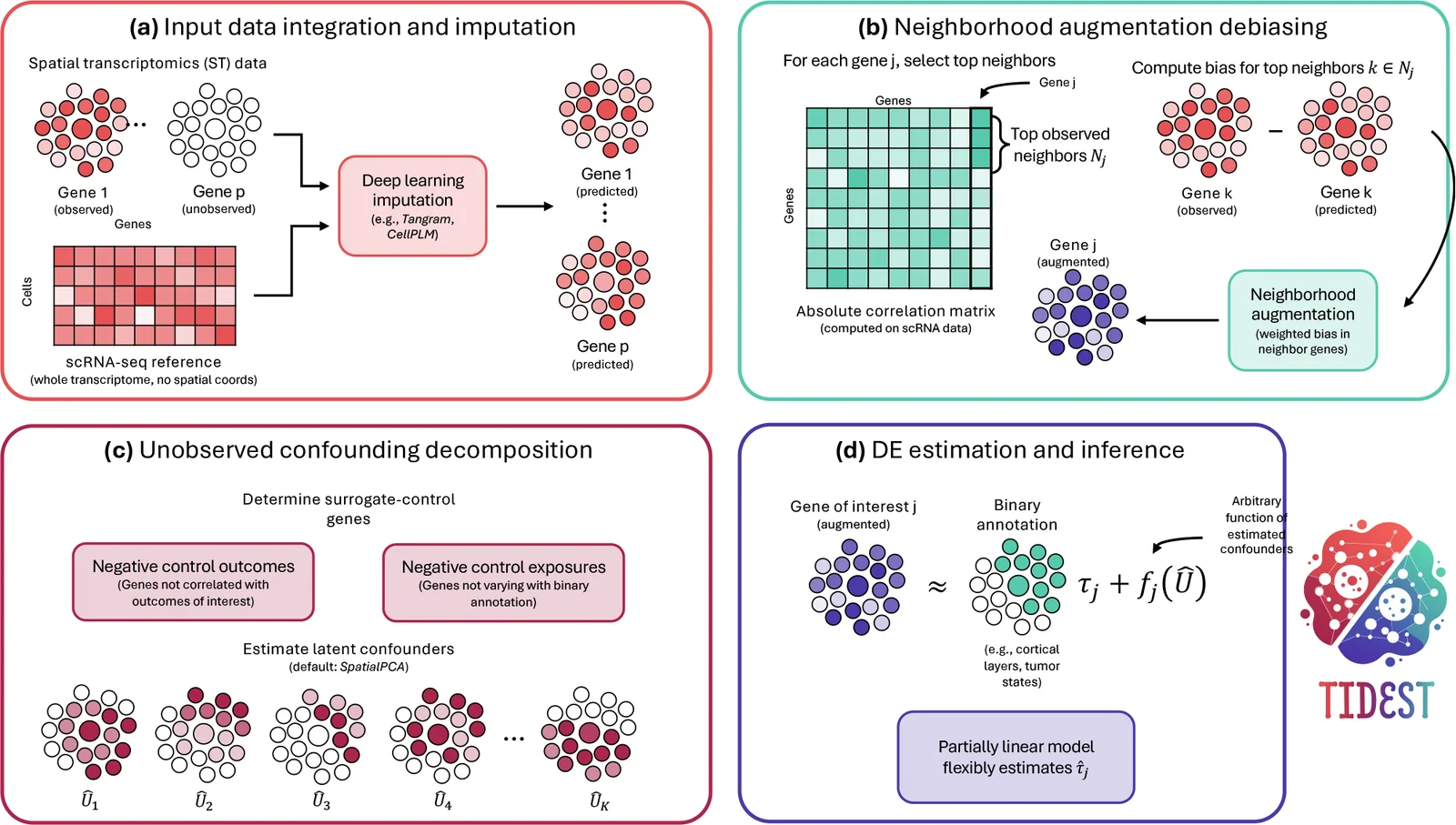
Overview of TIDEST. **(a)** TIDEST takes as input a spatial transcriptomics (ST) dataset, a matched scRNA-seq reference and the reconstructed transcriptome generated by an imputation model. The scRNA-seq reference provides transcriptome-wide information, whereas the ST data provide spatial localization. **(b)** TIDEST refines the reconstructed expression profiles using information from measured genes. For each target gene, the method identifies highly correlated observed genes and uses their prediction errors to correct systematic biases in the reconstructed expression. This produces an augmented expression profile that is less sensitive to errors introduced during imputation. **(c)** TIDEST identifies large-scale spatial patterns that may influence gene expression independently of the biological comparison of interest. Examples include anatomical structure, cell-type composition, microenvironmental gradients, and technical effects. These latent patterns are estimated from a set of control genes and summarized as a low-dimensional representation of spatial variation. **(d)** TIDEST performs differential expression testing between predefined biological groups while accounting for the estimated spatial variation. The output consists of effect estimates, confidence intervals, and significance measures for both observed and reconstructed genes, enabling transcriptome-wide DE analysis from spatially reconstructed transcriptomes.

TIDEST is a framework for transcriptome-wide differential expression testing in spatial transcriptomics datasets where some genes are reconstructed from a scRNA-seq reference. The workflow takes as input a ST dataset, a matched scRNA-seq reference, and the predicted spatial expression generated by an imputation model (Fig. 1a). TIDEST then refines the reconstructed expression profiles using information from measured genes (Fig. 1b), identifies latent spatial variation that may confound biological comparisons (Fig. 1c), and performs DE testing between predefined biological conditions (Fig. 1d). The output is a set of effect estimates, confidence intervals, and significance measures for both observed and reconstructed genes, enabling transcriptome-wide DE analysis while accounting for uncertainty introduced during spatial reconstruction.

The first stage addresses prediction uncertainty via a *debiasing* procedure (Fig. 1b) [27, 28]. Common practice treats imputed values ${\tilde{Y}}_{ij}$ for spatial coordinate i and gene j as true observations, ignoring the error introduced during reconstruction. TIDEST instead uses the observed residuals of highly correlated measured genes to construct an *augmented* outcome ${\hat{Y}}_{ij}$ for each target gene (see Methods), reducing systematic prediction errors and improving robustness to biases introduced by the underlying imputation model.

The second stage addresses unobserved confounding by estimating latent sources of variation from surrogate-control outcomes (Fig. 1c). In spatial transcriptomics, factors other than the biological comparison of interest can create apparent expression differences between groups. For example, anatomical structure, cell-type composition, microenvironmental gradients or technical effects may vary across a tissue and influence gene expression independently of the condition being studied [29]. TIDEST estimates these latent patterns (by default using SpatialPCA [30]) and incorporates them into downstream analyses, reducing the risk that such factors are mistaken for true differential expression signals.

The final stage performs DE testing while accounting for the estimated latent variation (Fig. 1d). TIDEST fits a partially linear model [31] relating the augmented outcomes to the biological condition of interest while flexibly adjusting for the estimated spatial confounders. Because inference is performed on the augmented outcomes rather than the raw predictions, TIDEST simultaneously addresses prediction uncertainty and unobserved spatial variation, enabling more reliable identification of differentially expressed genes across biological conditions.

### Simulation study provides strong support for TIDEST

**Figure 2:**
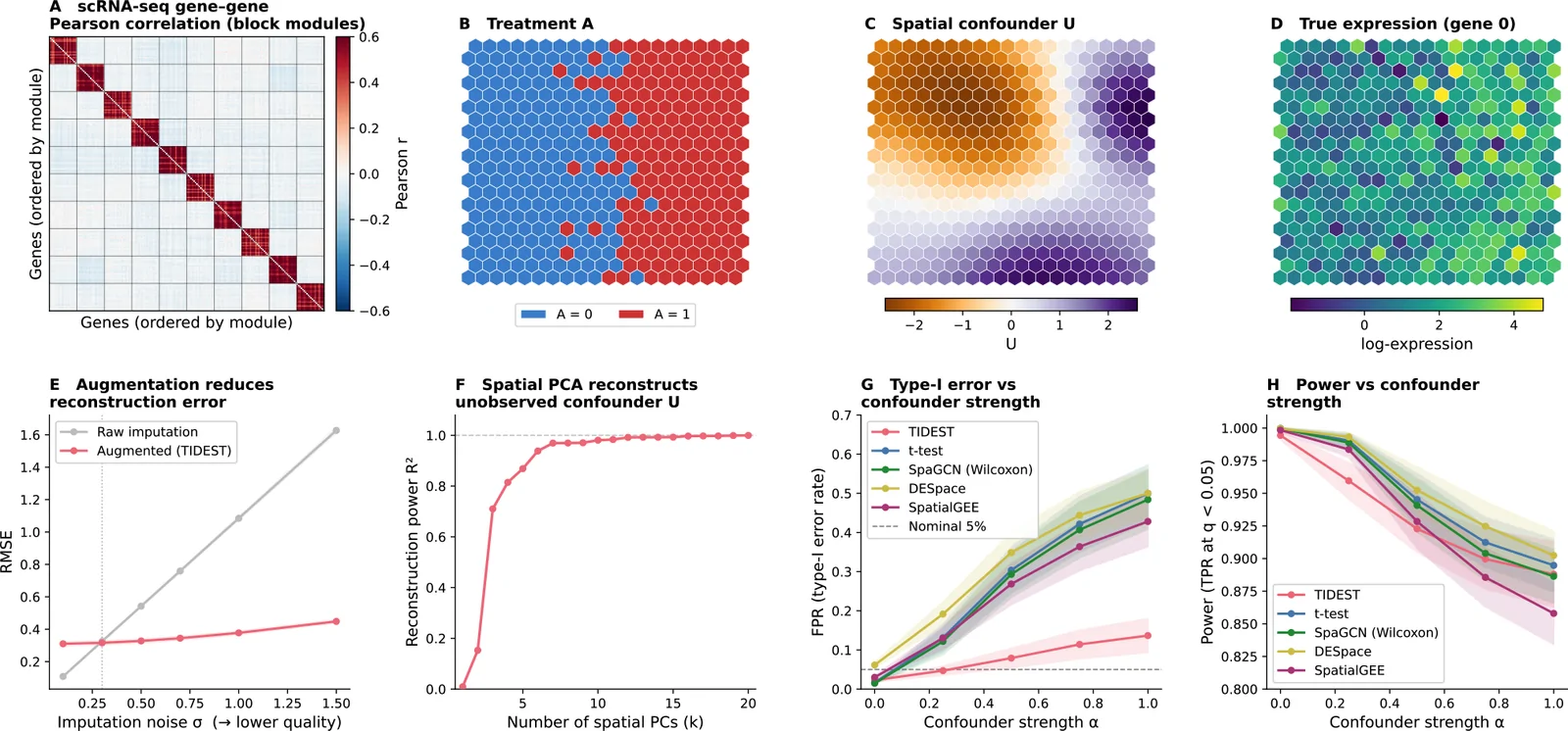
Simulation study validating TIDEST. **(a)** Block-diagonal Pearson correlation matrix of the synthetic scRNA-seq reference, computed from 2000 cells across 10 co-expression modules (200 genes). The block structure is the informative gene-gene correlation exploited by TIDEST augmentation strategy. **(b)** Binary treatment assignment A (two-region DGP, n=400 spots on a regular hexagonal grid). **(c)** Unobserved spatial confounder U, drawn as a Gaussian random field with length-scale ℓ=0.3. In this simulation setting, U is spatially smooth and independent of A, but its interaction with gene loadings $\beta_{j}$ induces systematic differences between treatment regions that naive comparisons absorb into the estimated treatment effect. **(d)** True log-expression of a representative DE gene, combining the treatment effect $\tau_{j}A_{i}$ and the confounder contribution $\alpha \beta_{j}U_{i}$. **(e)** Reconstruction quality of the augmented pseudo-outcome. RMSE of the raw imputed prediction ($\tilde{Y}$, gray) and the augmented outcome ($\overset{ˆ}{Y}$, red) against the true latent expression Y, as a function of imputation noise $\sigma_{\text{imp}}$ (lower $\sigma_{\text{imp}}$ means better imputer). The dashed vertical line marks the crossover $\left(\sigma_{imp}\approx 0.3\right)$ below which raw imputation is sufficient; above this threshold the augmented RMSE is bounded between 0.31 and 0.45 regardless of imputer quality. **(f)** Confounder reconstruction power. Coefficient of determination $R^{2}$ of U as a function of the number of spatial PCA eigenvectors k retained in the confounder matrix U, computed on the illustration replicate. Three eigenvectors capture 71% of the confounder variance; 10 eigenvectors reach 98%. **(g)** Type-I error rate (FPR at BH q<0.05) vs α (50 replicates per α level). The dashed line marks the nominal 5% level. All competing methods inflate FPR steeply with confounding; TIDEST maintains substantially lower FPR across all settings. Shaded bands are 95% confidence intervals across replicates. **(h)** Power (TPR at BH q<0.05) vs confounder strength α (50 replicates per α level).

We validated TIDEST on fully synthetic data in which ground-truth treatment effects, spatial confounders, and imputation errors are known by design (see Methods). An example scRNA-seq reference, spatial treatment A, unobserved spatial confounder U, and observed gene expression Y are shown in Fig. 2a–d. We compared TIDEST against a two-sample t-test and three representative methods for differential expression analysis in spatial transcriptomics [23]: SpaGCN [24] (Wilcoxon rank-sum test, also used in Seurat [32]), DESpace [25] (negative binomial generalized linear model), and SpatialGEE [26] (generalized estimating equations).

Augmentation provides no benefit when prediction is already highly accurate, but substantially improves reconstruction throughout the error range typical of current prediction methods (Fig. 2e). Importantly, augmentation remains stable across a wide range of imputation quality, indicating that downstream inference need not depend critically on the accuracy of the prediction model. The learned confounder representation $\overset{ˆ}{U}$ approximates the unobserved field U well (Fig. 2f) – even three eigenvectors capture most confounder variation, and increasing k yields near-complete removal of spatial confounding.

Existing methods rapidly lose false-discovery control as spatial confounding increases, whereas TIDEST remains substantially more robust (Fig. 2g). At α=1, all competing methods exhibit approximately ten-fold inflation in false discoveries (43–50% FPR), whereas TIDEST limits FPR to 13.7%. We next examine sensitivity (TPR at BH q<0.05): at α=0 all methods achieve near-perfect power, and at α=1.0 power converges toward 85–90% across methods (Fig. 2h). However, the apparent advantage of the t-test and DESpace [25] over TIDEST at high α is an artifact of inflated FPR – the BH threshold admits more false positives while true positives barely increase. This is evident from the AUC (Supp. Fig. B.1), which evaluates how well each method separates truly DE genes from null genes across all possible significance thresholds. At α=0 all methods achieve AUC ≈ 1, while at α=1.0 TIDEST achieves a 10 percentage-point advantage.

Together, these simulations demonstrate that accurate reconstruction does not guarantee valid biological inference. Rather, prediction uncertainty interacting with latent spatial variation produces systematic false discoveries unless explicitly modeled. By calibrating reconstructed expression and adjusting for latent spatial variation, TIDEST restores reliable differential-expression inference across a wide range of imputation quality.

### Correcting reconstruction bias resolves misleading laminar differential expression in the mouse neocortex

**Figure 3:**
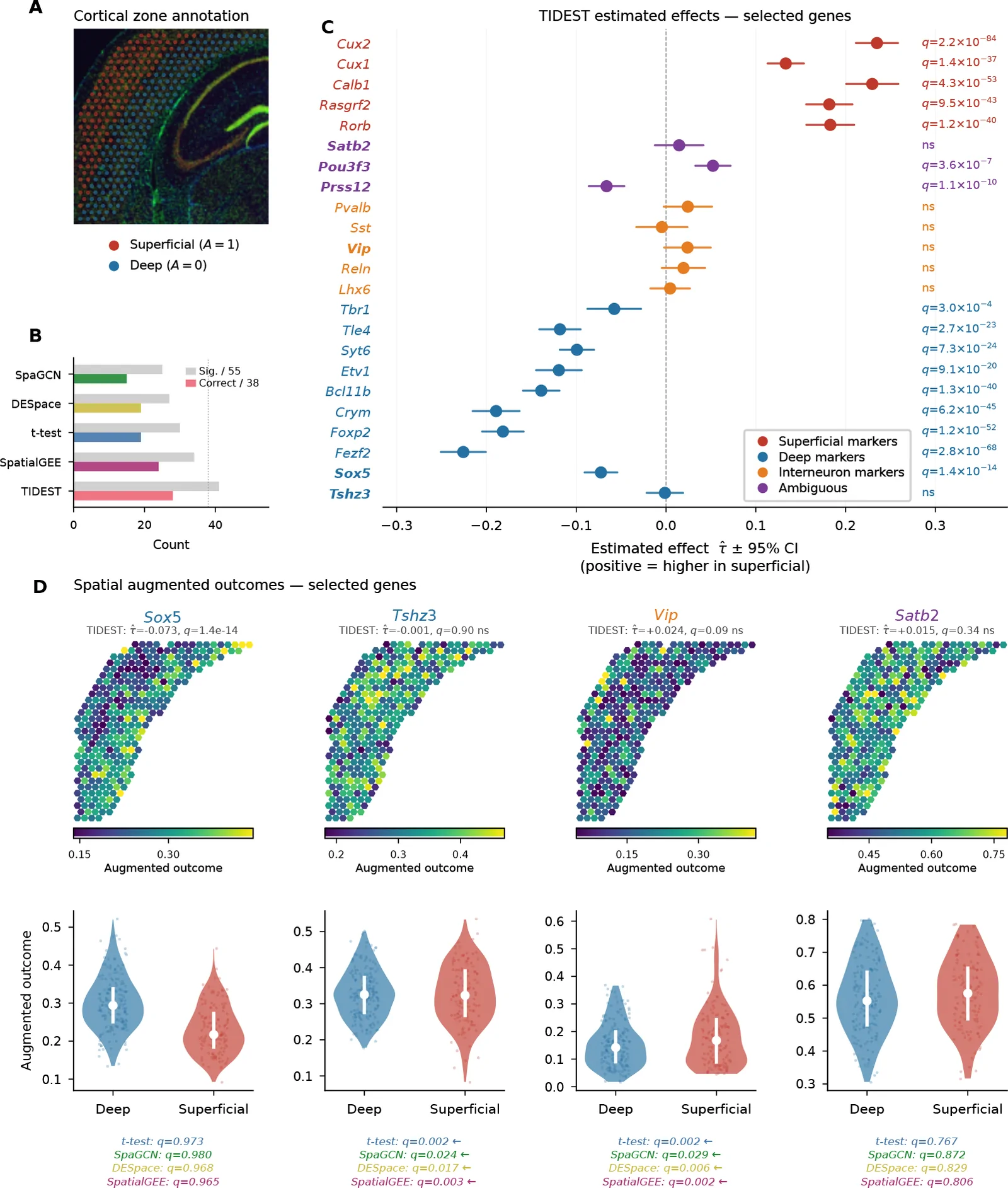
Mouse brain application. **(a)** Visium section of the adult mouse neocortex with cortical zone annotation overlaid as colored hexagonal spots (red: superficial layers L2/3/4, A=1; blue: deep layers L5/6, A=0; 297 spots total). **(b)** Method comparison summary. Grouped bars show, for each method, the number of genes called significant out of all 55 (gray) and the number that are both significant and correctly directioned out of the 38 genes with an established expected direction (colored). TIDEST achieves the highest correct count (28/38). **(c)** TIDEST estimated effects ($\overset{ˆ}{\tau }\pm 95\%CI$) for 23 selected genes. Color indicates gene category: red = superficial markers, blue = deep markers, orange = interneuron markers (negative controls), purple = ambiguous annotation. BH-adjusted q-values are shown to the right of each estimate. **(d, top row)** Spatial augmented outcome maps for four key genes (*Sox5*, *Tshz3*, *Vip*, *Satb2*). The subtitle reports the TIDEST estimate and BH-adjusted q-value. **(d, bottom row)** Deep vs. superficial augmented outcome distributions for the same four genes. Violins show the full distribution; the white bar marks the interquartile range; the white dot marks the median. BH-adjusted q-values for all four competing methods (SpaGCN, DESpace, t-test, SpatialGEE) are annotated below each violin. *Sox5*: TIDEST detects a deep signal ($q=1.4\times 10^{-14}$) that all competitors miss (q≥0.97). *Tshz3*: TIDEST returns a null result (q=0.90) while all competitors produce significant estimates in the wrong biological direction (q≤0.024). *Vip*: interneuron marker that may not differ by layer; TIDEST is non-significant (q=0.09) while all competitors produce significant results (q≤0.029). *Satb2*: expressed across both layers; all methods agree on non-significance.

We first evaluated whether TIDEST could improve the biological interpretation of reconstructed spatial transcriptomes in the adult mouse neocortex. We analyzed a Visium spatial transcriptomics section [33] comprising 297 annotated spots from superficial (L2/3/4; n=126) and deep (L5/6; n=171) cortical layers (Fig. 3a). To mitigate Visium dropout, we projected a matched scRNA-seq reference (21697 cells) onto the tissue using Tangram [10, 34]. Because cortical gene expression varies continuously across depth [35, 36], TIDEST accounted for this spatial structure when testing differential expression between superficial and deep layers (see Methods).

Several genes illustrate how accounting for reconstruction bias and latent spatial variation changes biological interpretation (Fig. 3d). *Sox5*, a canonical deep-layer marker [37, 38], is detected by TIDEST ($\overset{ˆ}{\tau }=-0.073,\;q=1.4\times 10^{-14}$) but missed by all four competitors (q≥0.965). Conversely, *Tshz3*, a deep-layer gene [39], is returned as non-significant by TIDEST (q=0.903), while all four competing methods instead identified significant differential expression in the opposite biological direction: t-test calls it superficial (q=0.002), as do SpatialGEE (q=0.003), DESpace (q=0.017), and SpaGCN (q=0.024). The clearest specificity advantage concerns interneuron markers, which are distributed across cortical layers and may not systematically differ between zones. *Vip*, a canonical interneuron marker [40], is called significant by every competitor (t-test q=0.002, SpatialGEE q=0.002, DESpace q=0.006, SpaGCN q=0.029), but TIDEST returns q=0.090 (non-significant). These examples illustrate that statistically valid inference changes biological interpretation rather than simply increasing statistical power.

To evaluate this improvement systematically, we assembled a curated panel of 55 cortical genes comprising 38 established layer markers with known directionality, five interneuron markers expected to show no laminar bias, and 12 genes with uncertain layer preference. Overall, TIDEST identified 41/55 (74.5%) as significantly differentially expressed (BH q<0.05) (Fig. 3b). The strongest superficial signal was *Cux2* ($\overset{ˆ}{\tau }=+0.235,\;q=2.2\times 10^{-84}$), a well-established upper-layer marker [41]; the strongest deep signal was *Fezf2* ($\overset{ˆ}{\tau }=-0.226,\;q=2.8\times 10^{-68}$), a canonical regulator of corticospinal neuron identity [42, 43] (Fig. 3c; further examples in Supp. Material). Compared with four competitors on the same gene panel (Fig. 3b; Supp. Fig. C.3), TIDEST recovered the most correct-direction significant genes (28 of 38), versus SpatialGEE (24), t-test (19), DESpace (19), and SpaGCN (15). The improvement was most apparent for genes with moderate effect sizes or gradual laminar gradients, where explicitly modeling spatial dependence reduced both false negatives and biologically misleading discoveries.

TIDEST also recovered signals consistent with recent transcriptomic evidence that refines earlier functional annotations (Fig. 3c); two further examples (*Pou3f3*, *Prss12*) are detailed in the Supp. Material. *Satb2* was originally characterized as a superficial marker required for callosal projection neuron identity in L2/3 [44, 45], although its immunoreactivity has also been observed in L6, challenging a strictly superficial interpretation [46, 47]. TIDEST returns a non-significant estimate (q=0.339; Fig. 3d), consistent with expression across both zones – a null result shared by all competing methods. Likewise, the remaining four interneuron markers were non-significant in TIDEST (Supp. Table C.1), suggesting that apparent layer associations detected by competing methods may instead reflect local interneuron clustering rather than robust laminar enrichment.

### Genetic risk for neuropsychiatric and cognitive traits converges on Layer 2/3 of the human DLPFC

**Figure 4:**
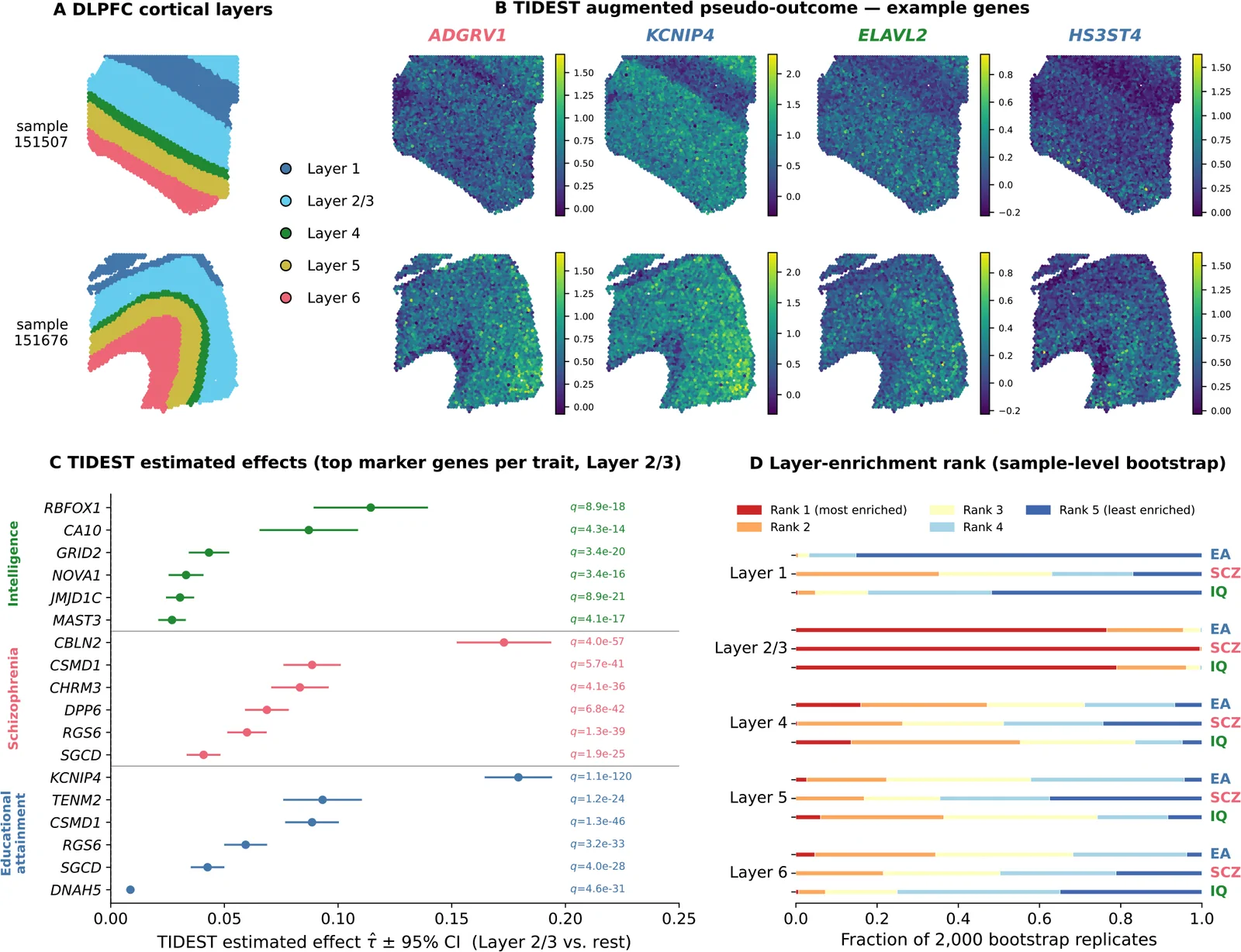
Human brain application. **(a, b)** Manual cortical-layer annotation (**a**) and TIDEST augmented spatial expression maps for four representative marker genes (**b**; *ADGRV1*, *KCNIP4*, *ELAVL2*, *HS3ST4*) across two representative DLPFC sections (151507, 151676), illustrating that GWAS-nominated marker genes for different traits peak in different cortical layers. **(c)** TIDEST estimated effects ($\overset{ˆ}{\tau }\pm 95\%\;CI$, Layer 2/3 vs. all other retained layers) for the top 6 marker genes (by pooled q-value) for each of three GWAS traits – educational attainment (EA), schizophrenia (SCZ), and intelligence (IQ) – fitted independently in each of the 12 DLPFC sections and combined by DerSimonian-Laird random-effects meta-analysis. q-values (BH-adjusted within Layer 2/3) are shown to the right of each estimate. **(d)** Stability of the layer ranking by trait-gene enrichment. For each trait and cortical layer, we resampled the 12 sections with replacement (2000 bootstrap replicates), recomputed the random-effects meta-analysis, and ranked the 5 layers by the common language effect size comparing trait genes to matched background genes (restricted to genes with positive $\overset{ˆ}{\tau }$). Stacked bars show, for each layer and trait, the fraction of the 2000 replicates in which that layer was assigned each rank (rank 1 = most enriched, rank 5 = least enriched). Layer 2/3 is the most consistently enriched layer for all three traits.

We applied TIDEST to 12 Visium sections of the human dorsolateral prefrontal cortex (DLPFC) from three neurotypical donors [48], asking whether genes nominated by GWAS Locus-to-Gene (L2G) analyses for three brain-related traits – educational attainment (EA) [49], schizophrenia (SCZ) [50], and intelligence (IQ) [51] – show layer-specific spatial enrichment relative to expression-matched background genes (Fig. 4a,b). We tested each of five manually annotated cortical layers (Layer 1, Layer 2/3, Layer 4, Layer 5, Layer 6) against all remaining retained layers and combined section-specific estimates across the 12 sections using random-effects meta-analysis (see Methods).

A strikingly consistent pattern emerged across all three traits: Layer 2/3 showed the strongest enrichment of GWAS-nominated genes. More trait genes reached genome-wide-adjusted significance (BH q<0.05) in Layer 2/3 than in any other layer, comprising 117/199 (58.8%) genes for EA, 141/221 (63.8%) for SCZ, and 78/146 (53.4%) for IQ (Fig. 4c). This convergence was observed despite the three trait panels being derived from non-overlapping discovery GWAS with distinct, though correlated, genetic architectures and independently constructed expression-matched background panels.

To determine whether Layer 2/3 enrichment represented a robust cross-section pattern rather than being driven by a small number of influential tissue sections, we performed a sample-level bootstrap. We resampled the 12 sections with replacement, recomputed the random-effects meta-analysis, and ranked the five layers by the common-language effect size comparing trait genes with expression-matched background genes (restricted to positive $\overset{ˆ}{\tau }$ values) across 2000 replicates. Layer 2/3 was ranked as the most enriched layer in 76.6% of replicates for EA, 99.6% for SCZ, and 79.0% for IQ (Fig. 4d), making it the modal top-ranked layer by a wide margin for every trait. Conversely, Layer 1 was consistently identified as the least-enriched layer for EA and IQ (85.2% and 51.8% of replicates ranked it last, respectively), whereas for SCZ the least-enriched rank was more evenly distributed between Layers 4 and 5. Together, these results identify Layer 2/3 as a reproducible point of convergence for genetic signals associated with cognitive and neuropsychiatric traits in the human DLPFC.

The strongest Layer 2/3-enriched gene differed across phenotypes, suggesting that this cross-trait convergence encompasses distinct molecular programs. For EA, the strongest signal was *KCNIP4* ($\overset{ˆ}{\tau }=0.179,\;q=1.1\times 10^{-120}$), which encodes a calcium-binding subunit of native Kv4 potassium channel complexes involved in neuronal excitability [52, 53]. For SCZ, *CBLN2* ($\overset{ˆ}{\tau }=0.173,\;q=4.0\times 10^{-57}$) encodes a synaptic organizer implicated in schizophrenia and related neuropsychiatric disorders [54, 55]. For IQ, *RBFOX1* ($\overset{ˆ}{\tau }=0.114,\;q=8.9\times 10^{-18}$) encodes a neuronal splicing factor regulating transcripts involved in neuronal excitability and has been linked to learning and memory [56]. Thus, independent genetic architectures converge spatially on Layer 2/3 while implicating distinct genes involved in neuronal excitability, synaptic organization, and RNA regulation.

### Spatial confounding obscures tumor-intrinsic transcriptional programs at the invasive margin of human glioblastoma

IDH-wildtype glioblastoma (GBM) is a genetically defined, highly heterogeneous primary brain tumor in which malignant cells coexist with and infiltrate normal brain tissue, complicating the interpretation of spatial expression differences. The cellular tumor (CT) and leading edge (LE) provide a particularly stringent setting for spatial differential expression: CT regions are densely packed with neoplastic cells, whereas LE regions contain infiltrating tumor cells interdigitated with resident brain tissue. Consequently, expression differences between these compartments can reflect both tumor-intrinsic transcriptional programs and changes in local cellular composition, making it difficult to distinguish malignant programs from signals contributed by the surrounding brain microenvironment.

**Figure 5:**
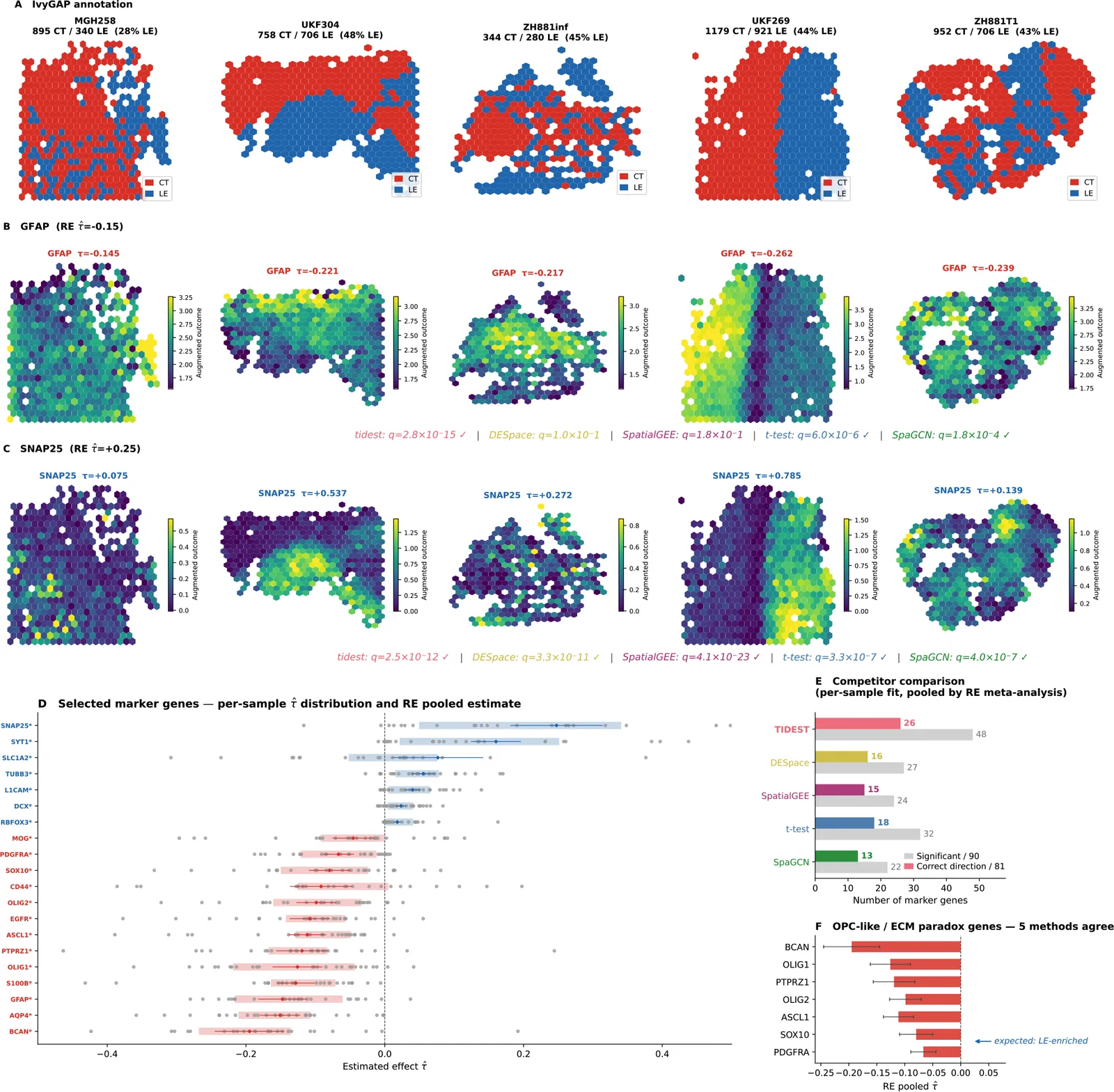
Human glioblastoma application. **(a–c)** IvyGAP histological annotation (**a**; CT in red, LE in blue) and augmented spatial expression maps of *GFAP* (**b**) and *SNAP25* (**c**) across five representative GBM sections (MGH258, UKF304, ZH881inf, UKF269, ZH881T1), illustrating the spatial cell-composition gradient that TIDEST disentangles from genuine tumor biology. Per-sample $\overset{ˆ}{\tau }$ values are shown above each map; pooled random-effects estimates are given in the row titles. Colored annotations below the maps report each method’s q-value for that gene, illustrating TIDEST’s power advantage on representative markers. **(d)** Per-sample $\overset{ˆ}{\tau }$ estimates (dots) and random-effects pooled estimates with 95% confidence intervals (diamonds and shaded bands) for a curated panel of marker genes, ordered by pooled $\overset{ˆ}{\tau }$ from strongly cellular-tumor (CT)-enriched (left, red) to strongly leading-edge (LE)-enriched (right, blue). **(e)** Number of the 90 panel genes called significant (BH q<0.05; gray bars) and, of the 81 genes with an *a priori* expected CT/LE direction, the number called significant with the correct sign (colored bars), for TIDEST and four competing methods (t-test, SpaGCN, DESpace, SpatialGEE), each fitted independently per sample across all 26 sections and pooled with the same DerSimonian-Laird random-effects meta-analysis. **(f)** Random-effects pooled $\overset{ˆ}{\tau }$ (with 95% confidence intervals) for the seven OPC-like and ECM “paradox” genes (*BCAN*, *OLIG1*, *PTPRZ1*, *OLIG2*, *ASCL1*, *SOX10*, *PDGFRA*). All seven are robustly CT-enriched ($\overset{ˆ}{\tau }<0$, BH $q<10^{-6}$ for every gene) and all five tested methods agree unanimously on the CT-enriched sign for every gene.

We therefore analyzed 26 10x Visium sections spanning three independent patient cohorts [57]. We fitted TIDEST independently to each section using the Ivy Glioblastoma Atlas Project (IvyGAP) annotation [58], comparing spots assigned to cellular tumor (CT) with those at the leading edge (LE), with positive $\overset{ˆ}{\tau }$ values indicating higher expression at the invasion front (Fig. 5a). To reduce the impact of dropout, expression was imputed using Tangram [10] with an external scRNA-seq reference from a disjoint cohort [59]. We assembled a curated panel of 90 genes (Supp. Table D.2) spanning GBM cell states, neuronal and myelin markers, invasion mediators, immune genes, and metabolic programs, and combined section-specific estimates using random-effects meta-analysis.

TIDEST resolved a clear transcriptional contrast between the tumor core and invasive margin. Among established GBM driver genes, *EGFR* – amplified in approximately 40% of IDH-wildtype tumors – showed robust CT enrichment ($\overset{ˆ}{\tau }=-0.108,\;q=2.3\times 10^{-12}$, significant in 73% of samples), demonstrating recovery of the expected spatial localization of a canonical genetic driver despite extensive cellular heterogeneity. *CD44*, one of the best-established mesenchymal GBM markers, was similarly CT-enriched ($\overset{ˆ}{\tau }=-0.092,\;q=1.4\times 10^{-4}$, 77%) [60]. The strongest CT-enriched signals corresponded to genes characteristic of the astrocyte-like transcriptional state identified in single-cell analyses of genetically diverse GBMs [61], including *AQP4* ($\overset{ˆ}{\tau }=-0.151,\;q=2.4\times 10^{-22}$, 85%), *S100B* ($\overset{ˆ}{\tau }=-0.128,\;q=4.0\times 10^{-18}$, 92%), and *GFAP* ($\overset{ˆ}{\tau }=-0.147,\;q=2.8\times 10^{-15}$, 73%), consistent with the high abundance of astrocyte-like malignant cells in the tumor core [62] (Fig. 5b).

In contrast, the strongest LE-enriched signals reflected the distinct biology of the invasive margin. The synaptic vesicle genes *SYT1* and *SNAP25* were among the most prominent positive-$\overset{ˆ}{\tau }$ signals (*SYT1*: $\overset{ˆ}{\tau }=+0.160,\;q=5.7\times 10^{-17}$, 73%; *SNAP25*: $\overset{ˆ}{\tau }=+0.247,\;q=2.5\times 10^{-12}$, 77%; Fig. 5c), and several additional neuronal and astrocytic markers were similarly LE-enriched (Supp. Material), consistent with the contribution of resident brain cells to this compartment. At the same time, *L1CAM*, a mediator of GBM migration along white matter tracts [63], emerged as a strong LE signal, indicating that the leading-edge profile is not explained solely by contamination from normal tissue but also captures biology associated with tumor invasion. Together, these patterns distinguish tumor-intrinsic programs concentrated in the cellular tumor from transcriptional signals arising at the interface between infiltrating malignant cells and surrounding brain tissue.

We next compared TIDEST with four published methods across the complete 90-gene panel, fitting each method independently to all 26 GBM sections and combining section-specific estimates using DerSimonian-Laird random-effects meta-analysis (BH q<0.05) (Fig. 5e). TIDEST recovered approximately 1.5–2× more significant genes than competing approaches while maintaining comparable direction accuracy among significant calls. DESpace and SpatialGEE, which explicitly model spatial structure, modestly improved direction accuracy over the naive t-test and SpaGCN, further underscoring the importance of spatial structure in this setting.

A notable feature of the data was that two classes of genes previously predicted from single-cell studies to be LE-enriched were instead consistently CT-enriched in the Visium data (Fig. 5f) [61]. The first comprised OPC-like and NPC-like GBM state markers, including *ASCL1* ($\overset{ˆ}{\tau }=-0.111,\;q=8.9\times 10^{-15}$, significant in 88% of samples), *OLIG1* ($q=4.4\times 10^{-11}$, 81%), *OLIG2* ($q=8.2\times 10^{-11}$, 73%), *SOX10* ($q=5.0\times 10^{-7}$, 77%), and *PDGFRA* ($q=2.6\times 10^{-8}$, 58%). The second comprised invasion-associated extracellular matrix genes, including *BCAN* ($\overset{ˆ}{\tau }=-0.195,\;q=3.9\times 10^{-13}$, 92%) and *PTPRZ1* ($\overset{ˆ}{\tau }=-0.119,\;q=1.6\times 10^{-9}$, 88%). All four competing methods recovered the same CT-enriched direction for all seven genes (Supp. Table D.3), arguing against a method-specific artifact. Instead, the discrepancy likely reflects the 55-*μ*m resolution of Visium spots, which can mix tumor cells across spatial compartments. This observation distinguishes inferential uncertainty from measurement resolution: accounting for reconstruction error and spatial confounding can improve inference, but cannot recover cellular resolution absent from the underlying measurement.

### TIDEST redefines the transcriptional landscape of human breast cancer invasion

Progression from ductal carcinoma *in situ* (DCIS) to invasive breast carcinoma (IBC) is accompanied by extensive remodeling of tissue architecture, including disruption of the myoepithelial barrier, activation of stromal fibroblasts, and immune infiltration. Consequently, spatial expression differences between pre-invasive and invasive regions reflect not only malignant progression but also substantial changes in local cellular composition, creating a setting in which latent spatial variation can alter the inferred transcriptional programs of invasion.

**Figure 6:**
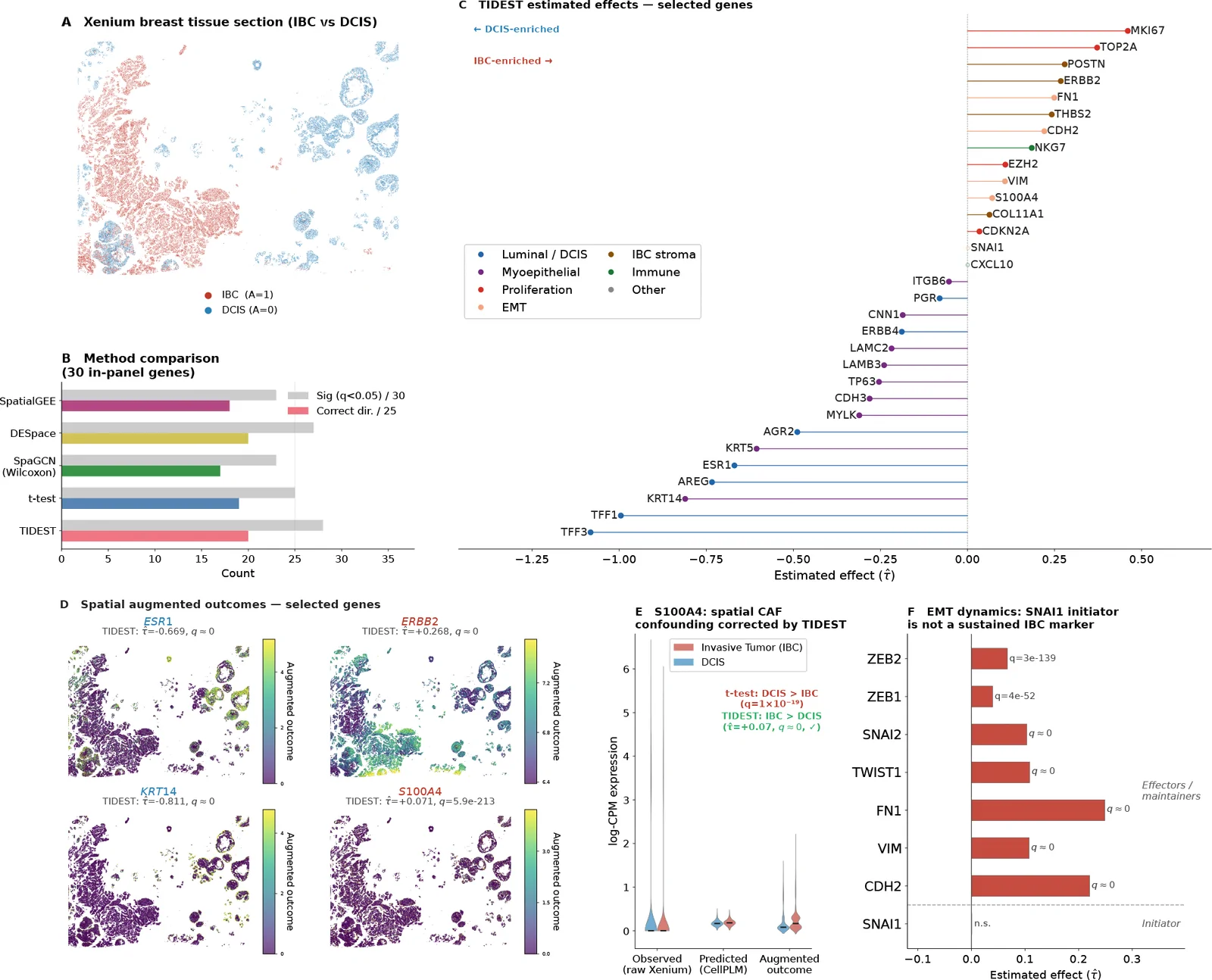
Human breast cancer application. **(a)** Xenium spatial transcriptomics section of human breast tissue (62755 cells). Each point is a single cell; red = IBC (A=1,n=28961); blue = DCIS (A=0,n=33794). **(b)** Method comparison on the 30 genes present in the observed Xenium panel. Grouped horizontal bars show, for each method, the number of genes called significant out of 30 (gray) and the number that are both significant and correctly directioned out of the 25 directional in-panel genes (red, TIDEST color). Dashed vertical line marks 25 (total directional genes). TIDEST and DESpace tie at 20/25 correct directions. **(c)** TIDEST estimated effects $\overset{ˆ}{\tau }$ for selected labeled marker genes, sorted by effect size (DCIS-enriched at left, IBC-enriched at right). Color encodes biological group (see legend). Dashed vertical line at $\overset{ˆ}{\tau }=0$. **(d)** Spatial augmented outcome maps for four key genes (*ESR1*, *ERBB2*, *KRT14*, *S100A4*). Gene name color encodes direction (red = IBC-enriched; blue = DCIS-enriched). Subtitle reports the TIDEST$\overset{ˆ}{\tau }$ and q-value. **(e)** *S100A4*: distributions of observed Xenium expression (left), CellPLM predicted expression (center), and augmented outcome (right), split by IBC (red) and DCIS (blue). Naive comparison using t-test on observed expression returns DCIS > IBC ($q=1\times 10^{-19}$, incorrect direction). TIDEST on the augmented outcome recovers the biologically expected IBC enrichment ($\overset{ˆ}{\tau }=+0.07,\;q\approx 0)$. **(f)** EMT dynamics. *SNAI1* (initiator) is non-significant ($\overset{ˆ}{\tau }\approx 0,\;q=0.15$); all seven downstream EMT effectors and structural remodeling markers are significantly IBC-enriched, consistent with a tumor that has completed rather than is currently undergoing mesenchymal transition.

We applied TIDEST to a Xenium single-cell spatial transcriptomics dataset of human breast tissue containing both DCIS and IBC within the same tissue section [3], analyzing 62755 annotated locations (Fig. 6a). We defined $A_{i}=1$ for IBC and $A_{i}=0$ for DCIS, so positive estimates correspond to higher expression in invasive regions. Because the Xenium panel measures only 313 genes, we reconstructed the transcriptome using CellPLM [21], a pre-trained cell language model, together with a matched scRNA-seq reference comprising 27000 cells [3], and used the reconstructed expression to construct the augmented outcomes.

Accounting for latent spatial variation altered the biological interpretation of several genes associated with invasion. The clearest example was the cancer-associated fibroblast marker *S100A4*, which has an established role in tumor invasion and is expected to be enriched in IBC [64]. The naive t-test identified *S100A4* as significantly differentially expressed in the opposite direction, with higher expression in DCIS. This reversal is consistent with fibroblasts interdigitated around DCIS ducts contributing disproportionately to local cellular composition and thereby biasing conventional comparisons. After adjustment with TIDEST, *S100A4* was recovered as IBC-enriched (Fig. 6e), demonstrating that latent spatial variation can invert the inferred direction of differential expression for biologically important invasion-associated genes. *NKG7* provides a second example of this phenomenon (Supp. Material).

TIDEST also refined the interpretation of regulatory programs governing invasion. *SNAI1*, a canonical initiator of epithelial-to-mesenchymal transition (EMT) that represses *CDH1* to induce cellular plasticity [65], was not significantly differentially expressed between DCIS and IBC (Fig. 6f). In contrast, downstream components of the EMT transcriptional network – including *CDH2*, *VIM*, *TWIST1*, *FN1*, *ZEB1*, and *ZEB2* – were all significantly enriched in invasive regions. This pattern distinguishes transient activation of an upstream regulatory factor from persistent activation of its downstream transcriptional program, consistent with an established invasive phenotype in which mesenchymal gene expression is maintained after *SNAI1* expression has subsided. Rather than indicating ongoing EMT induction, these data suggest that the invasive regions have progressed to a stable mesenchymal transcriptional state.

These changes in biological interpretation occurred within a broader transcriptional program consistent with progression from pre-invasive to invasive disease. We assembled a panel of 68 genes representing luminal differentiation, HER2 signaling, proliferation, EMT, extracellular matrix remodeling, immune infiltration, and myoepithelial integrity; 25 genes had well-established expected directionality in the IBC-versus-DCIS comparison. TIDEST identified 65 of 68 genes as significantly differentially expressed (BH q<0.05) (Fig. 6b). DCIS was enriched for canonical luminal differentiation genes, including *TFF3*, *TFF1*, *AREG*, *AGR2*, and the estrogen receptor *ESR1*. In contrast, IBC exhibited coordinated activation of proliferative, oncogenic, and invasive programs, including *MKI67*, *TOP2A*, the breast cancer driver *ERBB2*, the extracellular matrix regulator *POSTN*, and the mesenchymal marker *FN1* (Fig. 6c,d) [66].

We next compared TIDEST with competing methods on the 30 genes directly measured by the Xenium panel (Fig. 6b). Among the 25 directional markers, TIDEST recovered 20 with the expected direction, matching the best competing method while detecting the largest number of significant genes overall. Thus, the principal advantage of TIDEST in this setting was not simply increased detection, but the ability to distinguish transcriptional changes associated with invasion from differences arising from the spatial organization of the surrounding tissue.

Histological context also clarified the apparently paradoxical behavior of *KRT5* and *KRT14*, which were strongly enriched in DCIS despite frequently being described as basal-like breast cancer markers. In this spatial comparison, their expression is consistent with preservation of the myoepithelial layer surrounding DCIS ducts, which is lost following basement membrane invasion [67, 68]. This interpretation is supported by concordant enrichment of additional myoepithelial markers and was consistently recovered across competing methods (Supp. Material). Thus, apparent differences between pre-invasive and invasive regions can encode tissue architecture as well as tumor-cell state, reinforcing the importance of histological context when interpreting spatial differential expression.

## Discussion

Spatial transcriptomics is entering a new phase in which biological discovery increasingly depends on computationally reconstructed rather than directly measured transcriptomes. As reconstruction methods continue to improve, the challenge increasingly extends beyond prediction accuracy to the validity of downstream biological inference. Our results suggest that the next challenge is no longer reconstructing missing molecular measurements, but drawing valid biological conclusions from them. By treating reconstructed expression as experimentally observed, current analyses overlook both prediction uncertainty and the spatial organization of tissues, potentially producing misleading differential-expression signals and obscuring disease-associated transcriptional programs. This disconnect between prediction and inference represents an emerging challenge for computational spatial biology.

Across four biologically distinct systems, statistically valid inference consistently changed the interpretation of reconstructed spatial transcriptomes. In the mouse neocortex, it refined laminar transcriptional profiles by reducing biologically implausible differential-expression signals. In the human dorsolateral prefrontal cortex, it revealed a consistent convergence of genetic risk for educational attainment, intelligence and schizophrenia within Layer 2/3. In glioblastoma, it separated tumor-intrinsic transcriptional programs from the surrounding brain microenvironment at the invasive margin. In breast cancer, it redefined transcriptional programs associated with invasion by correcting misleading differential-expression calls and distinguishing persistent mesenchymal programs from transient regulatory activation. Together, these findings demonstrate that improving statistical inference does more than reduce false discoveries – it changes biological interpretation.

Several limitations warrant consideration. First, TIDEST depends on the availability of an appropriate single-cell reference, and performance may decline when the reference fails to capture the full cellular diversity represented in the spatial dataset. Second, the augmentation procedure assumes that prediction errors can be partially inferred from highly correlated observed genes; this assumption may be less reliable in settings with weak gene–gene correlation structure. Third, the current framework focuses on binary differential expression testing. Extending the methodology to continuous exposures, multiple experimental conditions, temporal trajectories, and hierarchical study designs represents an important direction for future research. Finally, variation in cell-type composition is a major determinant of spatial expression heterogeneity and may confound differential expression analyses. In the current implementation, such effects are absorbed into the estimated latent factors, enabling adjustment for broad compositional gradients without explicitly modeling cell identity. Nevertheless, integrating cell-type-specific information directly into the inferential framework may further improve power and interpretability.

Although developed for differential expression, the principles underlying TIDEST extend beyond this application. Prediction-powered calibration and statistically valid post-prediction inference may prove broadly useful for analyses of reconstructed molecular measurements, including gene-set enrichment, co-expression analysis and spatially variable gene detection. As computational reconstruction becomes routine across spatial omics, the central challenge will increasingly shift from predicting molecular measurements to interpreting them reliably. We anticipate that statistically valid inference will become an essential component of computational spatial biology, enabling increasingly accurate reconstruction to translate into reproducible biological discovery.

## Methods

### Details on TIDEST

*Testing for Imputed Differential Expression in Spatial Transcriptomics* (TIDEST) is a multi-stage framework designed to perform rigorous, spatially aware hypothesis testing on imputed spatial transcriptomics data. The pipeline addresses the dual challenges of prediction uncertainty – where downstream workflows naively treat noisy, deep learning-generated imputations as ground truth observations – and spatial autocorrelation, which violates standard sample independence assumptions and induces severe false-positive inflation in traditional statistical tests. To restore valid inference, TIDEST processes imputed expression matrices through three sequential modules: neighborhood augmentation debiasing, spatial gradient decomposition via surrogate-control outcomes, and orthogonalized DE effect estimation using partially linear models (PLMs).

To calibrate the systematic biases inherent in deep learning-based spatial reconstructions, TIDEST constructs a debiased augmented outcome ${\overset{ˆ}{Y}}_{ij}$ for each spatial coordinate i and target gene j. Rather than conducting downstream inference directly on the raw imputed values ${\tilde{Y}}_{ij}$, the framework leverages the empirical prediction residuals from a designated genomic neighborhood $N_{j}$ consisting of highly correlated, physically measured anchor genes. For a given target gene $j,\;N_{j}$ is populated by the most highly correlated, spatially observed genes (default: top 5 genes), where the feature-feature correlations ${\tilde{\sigma }}_{jk}$ are estimated from a scRNA-seq reference matching the biological system. Let $Y_{ik}$ denote the true observed expression of neighbor gene $k\in N_{j}$ at location i, and let ${\tilde{Y}}_{ik}$ be its corresponding model imputation. The debiased augmented outcome for the target gene is defined as:

(1) $${\overset{ˆ}{Y}}_{ij}={\tilde{Y}}_{ij}+\frac{1}{\left|N_{j}\right|}\sum_{k\in N_{j}}{\tilde{\sigma }}_{jk}\left(Y_{ik}-{\tilde{Y}}_{ik}\right).$$

By averaging the measured errors of observed spatial anchors, this procedure dynamically reduces the bias of unmeasured or sparsely captured transcripts exploiting information from ground-truth signals.

Spatially resolved transcriptomics data exhibit pervasive spatial autocorrelation, where large-scale tissue architectures (e.g., anatomical layering or tumor-microenvironment gradients) create continuous spatial patterns. When these structural variations correlate with a binary condition or tissue boundary, they act as unobserved confounders U, inflating Type-I error rates by violating standard independence assumptions. TIDEST addresses this by implementing an empirical spatial whitening procedure anchored on surrogate-control outcomes [69]. This strategy is closely related to surrogate variable analysis, where latent factors estimated from control features are used to account for unobserved variation [29]. In the present setting, these latent factors may represent spatial structure, cellular composition, technical artifacts, or other unmeasured sources of heterogeneity. To safely isolate local biological variation from confounding tissue-level trends, TIDEST scans the transcriptomic panel to identify a set of surrogate-control genes that satisfy two precise operational constraints:

- **Negative control outcome.** The empirical Pearson correlation between the target genes of interest and the candidate surrogate controls computed on the scRNA-seq reference falls below a threshold $r_{NCO}$.
- **Negative control exposure.** The spatial variability of the candidate surrogate controls – quantified by their coefficient of variation, defined as 100*γ/μ, where γ is the standard deviation and μ is the mean of a given spatial gene expression – falls below a threshold $r_{\text{NCE}}$.

These joint conditions guarantee that the selected surrogate-control genes are empirically decoupled from both the target biological outcomes and the primary treatment exposure. Once this control set is established, its spatial expression profile is utilized to reconstruct a low-dimensional representation of the latent confounding field $\overset{ˆ}{U}$. By default, TIDEST utilizes SpatialPCA to extract these latent principal components via a kernelized eigendecomposition [30], though alternative spatial dimension reduction techniques can be seamlessly integrated into the pipeline (e.g., StaNMF [70]).

In the final stage, TIDEST decouples the true treatment effect from large-scale spatial dependencies by mapping the augmented outcomes into a Robinson partially linear model (PLM) [31]. The conditional expectation of the debiased outcome is modeled as:

(2) $$E[{\overset{ˆ}{Y}}_{ij}|A_{i},{\overset{ˆ}{U}}_{i},X_{i}]=A_{i}\tau +f({\overset{ˆ}{U}}_{i},X_{i}),$$

where τ represents the core estimand of interest (the treatment effect of the binary condition A), $\overset{ˆ}{U}$ is the matrix of estimated confounders, and X captures additional spot-level covariates of interest (e.g., log-transformed library size). f(⋅) represents a flexible and unknown function mapping the spatial and technical confounders to expression levels.

By default, TIDEST utilizes random forests within a cross-fitting framework to flexibly estimate the nuisance functions in Equation (2). Crucially, this formulation yields an orthogonalized score function, guaranteeing that $\overset{ˆ}{\tau }$ achieves asymptotic normality and valid confidence intervals even when the non-parametric nuisance estimators or latent spatial components are estimated at slower, non-parametric rates [69].

### Simulation study design

We generated synthetic datasets consisting of n=300 spots on a regular two-dimensional grid, p=200 genes partitioned into 10 non-overlapping co-expression modules (20 genes each). We generated the binary treatment $A_{i}$ using the “two-region” DGP: treatment is a clean rectangular split – left half of the grid is assigned with A=0, right half has A=1. This is the most adversarial setting for naive methods because the spatial confounder U (a smooth Gaussian field) correlates strongly with the treatment boundary. In the Supp. Material, we explore additional DGPs. The true latent log-expression for gene j at spot i follows

(3) $$Y_{ij}=1+A_{i}\tau_{j}+\alpha U_{i}\beta_{j}+\varepsilon_{ij},$$

where $\tau_{j}∼𝒩\left(\tau_{\text{level}},0.1\tau_{\text{level}}\right)$ for the $p_{DE}=50$ differentially expressed genes, with random sign, and $\tau_{j}=0$ for the $p-p_{DE}=150$ null genes; $\beta_{j}∼𝒩(0,1)$ are gene-specific confounder loadings; $U_{i}∼𝒢𝒫\left(0,K_{\ell }\right)$ is a spatially smooth Gaussian random field with length-scale ℓ=0.3 that is independent of A;α∈{0,0.25,0.50,0.75,1.0} scales the confounder contribution; and $\varepsilon_{ij}∼𝒩(0,\;1)$. Observed counts are drawn as $C_{ij}∼Poisson\left(exp\left(Y_{ij}\right)\right)$.

The imputation model is parameterized as

(4) $${\tilde{Y}}_{ij}=Y_{ij}+\sigma_{imp}l_{j}^{⊤}\eta_{i}+\epsilon_{ij},$$

where $\eta_{i}∼𝒩\left(0,I_{M}\right)$ introduces module-structured noise, $l_{j}$ is the module loading vector of gene j (one entry is drawn from Uniform(0.5, 1.5), measuring the strength of its module membership; all the other entries are zero), and $\sigma_{\text{imp}}\in \{0.1,\;0.5,\;1.0,\;1.5\}$ parameterizes imputation quality (smaller means better). We also generate a synthetic single-cell reference of 2000 cells sharing the same module structure, so the Pearson correction exploits a realistic gene-gene correlation matrix.

We compared TIDEST against four spatial DE methods, all receiving identical observed count matrices and spatial coordinates: t-test (Welch two-sample t-test on log1p-normalized counts); SpaGCN [24] (Wilcoxon rank-sum test between spatial domains); DESpace [25] (negative-binomial model with spatial domain as a factor); and SpatialGEE [26] (generalized estimating equation with Poisson family and k-means working correlation structure).

In TIDEST pipeline, unobserved confounders were estimated using SpatialPCA [30] by truncated eigendecomposition of the Gaussian spatial kernel matrix retaining the top 20 eigenvectors. Eigenvectors with absolute Pearson correlation |r|>0.5 with the treatment A were dropped from the confounder matrix U prior to fitting. The PLM was fitted with two cross-fitting folds and 100 regression trees per nuisance estimator.

The main simulation grid crossed five confounder levels α∈{0,0.25,0.50,0.75,1.0} with three DGP variants and fixed $\tau_{\text{level}}=1.0,\;\sigma_{\text{imp}}=0.5$, yielding 15 settings; each was replicated 50 times. The DGP illustration panels (**A**-**D**) used n=400 spots on a hexagonal grid for visual clarity. The augmentation sweep (panel **E**) varied $\sigma_{\text{imp}}$ independently across six levels {0.1, 0.3, 0.5, 0.7, 1.0, 1.5} with 100 replicates per level, holding α=0 to isolate reconstruction quality from confounding. Extended results including all α levels and DGP-variant breakdowns are provided in the Supp. Material.

### Real-data processing

#### Mouse brain data

We used a mouse neocortex Visium section dataset [33], restricting to spots annotated as “Cortex_1” (deep layers L5/6, n=171) or “Cortex_3” (superficial layers L2/3/4, n=126; 297 spots total). We assembled a panel of 55 curated layer-marker genes comprising 15 genes expected to be superficial-enriched, 23 deep-enriched, and 17 additional markers (including cell-type markers and negative controls); expected directions were assigned from published layer-specific expression atlases [35, 71]. The binary treatment is $A_{i}=1$ for superficial spots and $A_{i}=0$ for deep spots, so a positive τ indicates higher superficial expression. The scRNA-seq reference was an adult mouse cortex dataset [34] (21697 cells), normalized to 10^4^ counts. Tangram [10] was fitted with RNA-count-based density prior; imputed expression was Pearson-augmented using the top-5 nearest-neighbors. SpatialPCA [30] was run retaining 10 principal components; one PC with absolute correlation |ρ|=0.779 with the treatment A was dropped by the correlation screen (threshold |ρ|>0.5), leaving nine SpatialPCA components as an estimate of unmeasured confounding. Log library size was appended as a covariate. The Robinson PLM was fitted with two cross-fitting folds and 200 regression trees per nuisance estimator (random-forest implementation). The four competing methods (t-test, SpaGCN Wilcoxon, DESpace, and SpatialGEE) were applied with identical settings to the simulation study, operating on the log1p-normalized observed Visium counts. All methods received the same 55 marker genes, and FDR control was applied independently within each method using the Benjamini-Hochberg procedure.

#### Human brain data

We analyzed 12 dorsolateral prefrontal cortex (DLPFC) 10x Visium spatial transcriptomics sections from three neurotypical donors [48]. Each section is annotated with manual cortical-layer labels; we merged Layer 2 and Layer 3 into a single “Layer 2/3” category for consistency across sections, retained only spots annotated as Layer 1, Layer 2/3, Layer 4, Layer 5, or Layer 6, and discarded spots annotated as white matter. For each of three GWAS traits under study – educational attainment (EA) [49], schizophrenia (SCZ) [50], and intelligence (IQ) [51] – we queried the Open Targets Platform Locus-to-Gene (L2G) pipeline for the top-predicted gene at each GWAS credible set, yielding 200 (EA), 221 (SCZ), and 146 (IQ) trait genes. Each trait gene was paired with 10 expression-matched background candidate genes (nearest neighbors in pooled observed log-CPM expression across the 12 sections, restricted to genes retained by Tangram in every sample), giving background panels of 2000 (EA), 2210 (SCZ), and 1460 (IQ) genes tested alongside the corresponding trait genes. As TIDEST requires a single-cell reference for imputation, we used a DLPFC snRNA-seq reference [72] (5231 nuclei from 2 neurotypical donors, disjoint from the 10x Visium cohort). The single-cell reference was normalized to 10^4^ counts. For Tangram [10] training we selected the top 100 marker genes per reference cell type (including layer-resolved excitatory subtypes), using the reference cell-type labels as the grouping variable. Tangram was fitted in cells mode with an RNA-count-based density prior.

The augmented outcome for each spot was constructed using the Pearson correlation correction with top-5 neighbors, with the Pearson correlation matrix computed from the snRNA-seq reference. We applied per-spot total-count scaling to align the Tangram imputed expression to the observed 10x Visium count scale before log-transformation. For each sample, we selected confounder genes for SpatialPCA as those with coefficient of variation $r_{NCE}<0.3$ in the augmented outcomes and Pearson correlation $\left|r_{\text{NCO}}\right|<0.3$ with any panel gene in the scRNA-seq reference. The top 2000 such genes ranked by spatial variance were used to fit SpatialPCA with 10 principal components. For each sample, SpatialPCA PCs with correlation with the treatment greater than 0.5 were excluded from the confounder matrix before fitting the PLM. Log-library size was appended as an additional covariate. For each of the 5 cortical layers, we fit the PLM independently for each of the 12 sections – treatment defined as a spot being annotated to that layer versus all other retained layers – using 2-fold cross-fitting and random forests with 200 trees. Per-sample p-values were adjusted by the Benjamini-Hochberg procedure within each sample (q<0.05).

Cross-sample meta-analysis used DerSimonian-Laird random-effects inverse-variance weighting to account for between-sample heterogeneity, followed by a second round of Benjamini-Hochberg correction over all genes tested within that layer. The between-sample variance ${\overset{ˆ}{\tau }}^{2}$ was estimated as ${\overset{ˆ}{\tau }}^{2}=max(0,(Q-(k-1))/c)$, where $Q=\sum_{i}w_{i}{\left({\overset{ˆ}{\tau }}_{i}-{\overset{ˆ}{\tau }}_{FE}\right)}^{2}$ is Cochran’s heterogeneity statistic, k=12, and $c=\sum_{i}w_{i}-\sum_{i}w_{i}^{2}/\sum_{i}w_{i}$ with $w_{i}=1/{\overset{ˆ}{\sigma }}_{i}^{2}$. To assess the stability of the layer ranking by enrichment, we performed a sample-level bootstrap (2000 replicates). In each replicate, for every cortical layer we resampled the 12 sections with replacement and recomputed the DerSimonian-Laird random-effects meta-analysis (as above) on the resampled sample set. Restricting to genes with positive ${\overset{ˆ}{\tau }}_{RE}$, we computed a rank-based effect size – the common language effect size (CLES), the probability that a randomly drawn trait gene’s ${\overset{ˆ}{\tau }}_{RE}$ exceeds that of a randomly drawn background gene, obtained from the Mann-Whitney U statistic – comparing the trait genes to all background candidate genes within that layer. The 5 layers were then ranked by this CLES value (rank 1 = most enriched, rank 5 = least enriched) for that replicate. Repeating this across all 2000 replicates gives, for each layer, an empirical distribution over its rank position, which we use to quantify how consistently each layer is identified as the most (or least) trait-enriched under resampling of the 12 sections.

#### Human glioblastoma data

We analyzed 26 IDH-wildtype GBM Visium spatial transcriptomics sections from three patient cohorts [57]: the Massachusetts General Hospital (MGH) cohort (n=1), the University of Freiburg (UKF) cohort (n=13), and the University Hospital Zurich (ZH) cohort (n=12). Each section is annotated with IvyGAP histological regions [58]; we retained only spots annotated as cellular tumor (CT) or leading edge (LE), discarding spots with other IvyGAP annotations. We assembled a curated panel of 90 genes (predicted) representative of the major GBM molecular programs, normal brain cell types, immune microenvironment, and metabolic states. As TIDEST requires a single-cell reference for imputation, we used the human GBM scRNA-seq dataset [59] (3589 cells from a disjoint cohort of patients). The single-cell reference was normalized to 10^4^ counts. For Tangram [10] training we selected the top 100 marker genes per reference cell-type, using the reference cell-type labels as the grouping variable. Tangram was fitted in cells mode with an RNA-count-based density prior.

The augmented outcome for each spot i was constructed using the Pearson correlation correction with top-5 neighbors. The Pearson correlation matrix was computed once from the scRNA-seq reference and shared across all 26 samples. We applied per-spot total-count scaling to align the Tangram imputed expression to the observed Visium count scale before log-transformation. For each sample, we selected confounder genes for SpatialPCA as those with coefficient of variation $r_{NCE}<0.5$ in the augmented outcomes and Pearson correlation $\left|r_{NCO}\right|<0.3$ with any panel gene in the scRNA-seq reference. The top 2000 such genes ranked by spatial variance were used to fit SpatialPCA with 50 principal components. For each sample, SpatialPCA PCs with correlation with the treatment greater than 0.5 were excluded from the confounder matrix before fitting the PLM. Log-library size was appended as an additional covariate. We fitted the PLM independently for each sample using 5-fold cross-fitting and random forests with 200 trees. Per-sample p-values were adjusted by the Benjamini-Hochberg procedure within each sample (q<0.05). Cross-sample meta-analysis used DerSimonian-Laird random-effects inverse-variance weighting (as above) to account for between-tumor heterogeneity, with k=26, followed by a second BH correction over the 90 panel genes.

#### Human breast cancer data

We used a Xenium FFPE spatial transcriptomics section of human breast tissue [3] containing both invasive breast cancer and ductal carcinoma *in situ* in the same specimen (62755 cells, 313-gene panel). Cluster annotations were taken from the companion single-cell reference [3]. The binary treatment was defined as $A_{i}=1$ for cells annotated as “Invasive_Tumor” or “Prolif_Invasive_Tumor” (IBC, n=28961) and $A_{i}=0$ for cells annotated as “DCIS_1” or “DCIS_2” (DCIS, n=33794). We assembled a panel of 68 curated marker genes spanning IBC proliferation and invasion drivers, DCIS luminal markers, myoepithelial keratins, EMT effectors and maintainers, immune infiltrate markers, and contested genes; 25 genes have a well-established expected direction in the IBC-versus-DCIS contrast, assigned from published IBC, DCIS, and myoepithelial expression studies [67, 73, 74]. The expected direction for *KRT5* and *KRT14* was set to DCIS, as these myoepithelial markers reflect the intact basement membrane present in DCIS rather than the basal subtype designation used in gene-panel studies. Genes with unclear expected directions due to ER/HER2-status dependence (e.g., *EGFR*, *FOXA1*, *MLPH*, *GATA3*) were excluded from the directional evaluation.

Because the Xenium panel covers only 313 genes, we constructed the augmented outcome using CellPLM [21], a pre-trained Transformer-based cell language model. The matched single-cell reference (27000 cells, 18085 genes) was normalized to 10^4^ counts per cell and log1p-transformed before input to CellPLM; the same normalization was applied to the Xenium ST data. CellPLM outputs expression in log-CPM space, so the Pearson correction is applied directly as the difference between observed and predicted log-CPM, without additional scaling. The top-5 nearest-neighbor Pearson correction was applied to construct the augmented outcome. Confounder genes were selected using coefficient of variation threshold $r_{\text{NCE}}<1.5$ and marker correlation threshold $\left|r_{\text{NCO}}\right|\leq 0.3$, retaining 2000 genes; the higher $r_{\text{NCE}}$ threshold (compared to the Visium applications) reflects the greater single-cell-level expression variability in Xenium data.

Due to the large cell count, fitting SpatialPCA directly would require constructing a 62755 × 62755 kernel matrix, which exceeds available memory. We instead fitted SpatialPCA on a stratified subsample of 10000 cells (5000 per group), retaining 50 principal components, and then projected all 62755 cells into the fitted SpatialPCA space. No SpatialPCA component with absolute Pearson correlation |ρ|>0.5 with the treatment A was found, so none was excluded from the confounder matrix; log library size (log-CPM total per cell) was appended as a covariate. The PLM was fitted with 2 cross-fitting folds and 200 regression trees per nuisance estimator, retaining 50 SpatialPCA components (after the correlation screen) as confounders. Sandwich standard errors were computed from the cross-fitted residuals.

The four competing methods were applied to the 30 genes present in the observed Xenium panel, operating on log-CPM-normalized Xenium counts. For DESpace, 13 cells with zero total counts across the marker panel were filtered before constructing the SpatialExperiment object, as the calcNormFactors step in edgeR produces undefined normalization factors for zero-library cells. For SpatialGEE, a stratified subsample of 20000 cells (10000 per group) was used. FDR control was applied within each method using the Benjamini-Hochberg procedure. TIDEST additionally tested the 38 genes accessible only through CellPLM augmentation; these were not evaluated for competitors.

## Data Availability

Mouse brain application data are available directly from the squidpy package [75] through the built-in functions datasets.visium_fluo_adata_crop() (10x Visium) and datasets.sc_mouse_cortex() (scRNA-seq). Human brain application spatial (10x Visium) data can be downloaded from the HumanPilot GitHub webpage at https://github.com/LieberInstitute/HumanPilot. Single-cell reference data for the human brain application are available at this GitHub link: https://github.com/LieberInstitute/10xPilot_snRNAseq-human. GWAS credible sets were retrieved using the Open Targets GraphQL API, accession numbers GCST006250 (intelligence), GCST90503210 (schizophrenia), and GCST90105038 (educational attainment). Human glioblastoma application spatial data (10x Visium) are available under the accession number GSE237183 (GEO) and also stored on Zenodo at https://doi.org/10.5281/zenodo.8105466. Single-cell reference data for the human glioblastoma application are available under the accession number GSE84465 (GEO). Human breast cancer application data are available under the accession code GSE243280 (GEO) and also stored on 10x Genomics website at https://www.10xgenomics.com/products/xenium-in-situ/preview-dataset-human-breast. All code to reproduce the entire analysis pipeline, including simulations and real-data applications, as well as code containing TIDEST package, is available at https://github.com/testalorenzo/tidest.
